# Structure-based Design and Optimization of Novel, Potent and Selective Covalent FGFR2/3 Inhibitors with a Tricyclic Core

**DOI:** 10.1021/acs.jmedchem.6c00585

**Published:** 2026-06-08

**Authors:** Xuzhi Lu, Zhaodi Tian, Xueqiang Li, Xiaojuan Chen, Jean Ching-Yi Tien, Yayuan Yang, Yue Yin, Qingrun Li, Weixue Huang, Fengtao Zhou, Jinwei Zhang, Xiaomei Ren, Dan Liu, Abigail J. Todd, Shuqin Li, Yongheng Chen, Arul M. Chinnaiyan, Shaohua Chang, Ke Ding, Zhen Wang

**Affiliations:** 1 International Cooperative Laboratory of Traditional Chinese Medicine Modernization and Innovative Drug Discovery of Chinese Ministry of Education (MOE), Guangzhou City Key Laboratory of Precision Chemical Drug Development, College of Pharmacy, Jinan University, 855 Xingye Avenue East, Guangzhou 511400, China; 2 State Key Laboratory of Chemical Biology and Shanghai Hongkong Joint Laboratory in Chemical Synthesis, Shanghai Institute of Organic Chemistry, Chinese Academy of Sciences, #345 Lingling Rd., Shanghai 200032, China; 3 State Key Laboratory of Structure-Based Drugs Design & Discovery of Ministry of Education, Shenyang Pharmaceutical University, Shenyang 110016, China; 4 KinoTeck Therapeutics Co., Ltd, 35 Sicheng Road, Tianhe District, Guangzhou, 510663, China; 5 Department of Oncology, NHC Key Laboratory of Cancer Proteomics & State Local Joint Engineering Laboratory for Anticancer Drugs, National Clinical Research Center for Geriatric Disorders, Xiangya Hospital, Central South University, Changsha, Hunan 410008, China; 6 Michigan Center for Translational Pathology, University of Michigan, Ann Arbor, Michigan 48109, USA; 7 Department of Pathology, University of Michigan, Ann Arbor, MI 48109, USA; 8 National Facility for Protein Science in Shanghai, Shanghai Advanced Research Institute, Chinese Academy of Science, Shanghai 201210, China; 9 Howard Hughes Medical Institute, University of Michigan, Ann Arbor, MI 48109, USA; 10 Rogel cancer Center, University of Michigan Ann Arbor, MI 48109, USA; 11 Department of Urology, University of Michigan, Ann Arbor, MI 48109, USA

## Abstract

Fibroblast growth factor receptors 2 and 3 (FGFR2/3) are attractive therapeutic targets in multiple human cancers. Here, we report the discovery of a series of tricyclic 1-(4-amino-5-ethynyl-8,9-dihydropyrazino[1′,2′:1,5]pyrrolo[2,3-*d*]pyrimidin-7(6*H*)-yl)prop-2-en-1-one derivatives as novel covalent FGFR2/3 inhibitors through structure-based design and optimization. The lead compound, **KNT-0919**, potently suppressed FGFR2- or FGFR3-driven Ba/F3 cells (IC_50_ = 1.1 nM and 0.20 nM), with reduced activity against Ba/F3-FGFR1 cells (IC_50_ = 17.1 nM) and parental Ba/F3 cells (IC_50_ > 1000 nM). In a panel of 416 kinases, **KNT-0919** selectively inhibited FGFR2/3 kinases, including clinically relevant mutants. Covalent modification of FGFR2/3 was confirmed by mass spectrometry and X-ray crystallography. Functionally, **KNT-0919** selectively suppressed the proliferation of FGFR2-dependent cancer cell lines, dose-dependently inhibited FGFR2 downstream signaling, and induced apoptosis in an FGFR2-dependent manner. Moreover, **KNT-0919** demonstrated favorable oral bioavailability (56%) in rats and achieved significant tumor growth inhibition in an SNU-16 gastric cancer xenograft model.

## Graphical Abstract

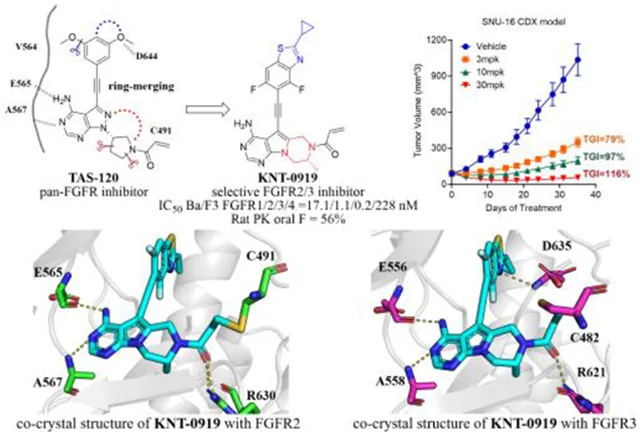

## INTRODUCTION

Fibroblast growth factor receptors (FGFRs) represent a family of four structurally related receptor tyrosine kinases (FGFR1-4) that play pivotal roles in regulating cell proliferation, differentiation, survival, and angiogenesis.^1,2^ These functions are primarily mediated through the activation of key downstream signaling cascades, including RAS-mitogen-activated protein kinase (MAPK), phosphatidylinositol 3-kinase (PI3K)–protein kinase B (AKT), and phospholipase C γ (PLCγ) pathways.^3–5^ Dysregulated FGFR signaling, frequently caused by genetic alterations such as gene amplification, activating mutations, and chromosomal translocations, is a well-established driver in the pathogenesis of numerous cancers.^6,7^ For instance, FGFR2 alterations are commonly found in cholangiocarcinoma, gastric cancer, and endometrial cancer,^8^ whereas FGFR3 alterations are predominantly associated with bladder cancer and multiple myeloma.^9^

The clinical validation of FGFR2/3 as therapeutic targets is evidenced by the U.S Food and Drug Administration (FDA) approval of several pan-FGFR inhibitors (*e.g.* futibatinib (TAS-120), erdafitinib, and pemigatinib) for cancers harboring FGFR2/3 alterations.^10–14^ However, the clinical utility of these agents is constrained by significant limitations: (1) dose-limiting toxicities linked to the inhibition of FGFR1 (causing hyperphosphatemia) and FGFR4 (leading to diarrhea);^15–17^ and (2) the emergence of on-target resistance mutations, particularly gatekeeper substitutions in the kinase domain (*e.g.*, FGFR2 V564F/I), which diminish drug binding affinity.^18–21^ To address these challenges, the selective targeting of FGFR2 and/or FGFR3 has emerged as a promising strategy for treating cancers driven by these specific isoforms.

Several selective FGFR2/3 inhibitors (**1-7**, Figure 1) have been developed, with a number advancing into clinical trials.^22–34^ Lirafugratinib (RLY-4008, **1**), developed by Relay Therapeutics, is a first-in-class, highly selective covalent FGFR2 inhibitor.^22,23^ Its design leverages the targeting of Cys491 within the relatively rigid P-loop of FGFR2, resulting in potent inhibition (IC_50_ = 3 nM) and exceptional selectivity over FGFR1 (>256-fold) and FGFR4 (>5000-fold). In Phase 1/2 trial (NCT04526106), compound **1** has demonstrated a 73% objective response rate in FGFRi-naïve FGFR2-fusion cholangiocarcinoma patients, with no clinically significant off-target adverse effects reported to date. LOXO-435 (LY3866288, **2**), from LOXO Oncology, is a highly selective FGFR3 inhibitor.^24,25^ It exhibits potent activity against FGFR3 (IC_50_ = 0.3 nM), with 361-fold and 66-fold selectivity over FGFR1 and FGFR2, respectively. Importantly, it retains efficacy against the clinically relevant FGFR3 V555M gatekeeper mutation (IC_50_ = 1.1 nM). Compound **2** has shown robust antitumor efficacy in preclinical models without inducing hyperphosphatemia and is currently being evaluated in a Phase 1 trial (NCT05614739) for advanced solid tumors harboring FGFR3 alterations. TYRA-300 (**3**), developed by Tyra Biosciences, is a reversible, selective FGFR3 inhibitor.^26^ It demonstrates potent FGFR3 inhibition (IC_50_ = 11 nM) and high selectivity over other isoforms (FGFR1: 25-fold; FGFR2: 14-fold; FGFR4: 36-fold). A critical feature is its maintained strong activity against gatekeeper mutants FGFR3 V555M (IC_50_ = 2.0 nM) and V555L (IC_50_ = 1.5 nM). Compound **3** is undergoing evaluation in Phase II trials (NCT06995677, NCT05544552) for FGFR3-altered urothelial carcinoma and other solid tumors. Together, these developments demonstrate the clinical value of selectively targeting FGFR2 and FGFR3.

Here, we report the discovery of a series of tricyclic tetrahydropyrazino–pyrrolo–pyrimidinylamino derivatives as novel covalent FGFR2/3 inhibitors *via* structure-based design and optimization. Among these, **KNT-0919** potently inhibited FGFR2 and FGFR3 kinase activity and selectively suppressed the growth of FGFR2- or FGFR3-driven cancer cells, including those harboring clinically relevant mutant variants. Notably, **KNT-0919** exhibited favorable oral bioavailability (56%) in rats and achieved significant tumor growth inhibition in an SNU-16 gastric cancer xenograft model.

## RESULTS AND DISCUSSION

### Molecular Design.

Our medicinal chemistry campaign commenced with Futibatinib (TAS-120), an approved irreversible pan-FGFR inhibitor developed by Taiho Oncology. This compound demonstrated potent enzymatic inhibition of FGFR1-4, with IC_50_ values ranging from 1.3 to 8.3 nM, and also inhibited the most commonly observed gatekeeper mutant FGFR2^V564F^ with an IC_50_ value of 83 nM. The co-crystal structure of TAS-120 bound to FGFR2 (PDB: 8W2X) was publicly available, which facilitated the design of new selective FGFR2/3 inhibitors. Analysis of this structure revealed that the pyrazolo[3,4-*d*]pyrimidine core forms hydrogen bonds with hinge residues Glu565 and Ala567, while its acrylamido warhead covalently targets Cys491 in the P-loop (Figure 2A). Additionally, the dimethoxyphenyl moiety extends into the hydrophobic back pocket, where one of the methoxy groups engages in a hydrogen-bond interaction with Asp644.

Inspired by prior work from Relay Therapeutics, which showed that differences in P-loop flexibility between FGFR1 and FGFR2 could be leveraged to enhanced FGFR2 selectivity over FGFR1,^22^ we sought to improve isoform selectivity by cyclizing the pyrazolyl and piperidyl moieties of TAS-120 to introduce structural rigidity into the acrylamide-containing region (Figure 2C). Furthermore, the predicted binding mode of TAS-120 with FGFR2^V564F^ indicated that the substitution of Val564 with Phe564 causes a steric clash with one methoxy group of TAS-120, likely impairing binding (Figure 2B). To avoid this clash, we removed one methoxy group and applied a ring-merging strategy aimed at engaging in π–π interactions with the nearby Phe645. Additionally, a nitrogen atom was introduced in the newly formed ring to maintain a key hydrogen bond with Asp644 (Figure 2C). The resulting compound, **8a**, was selected for docking studies against both wild-type FGFR2 and the V564F mutant. The docking results showed that in both wild-type FGFR2 and FGFR2^V564F^, all key interactions with **8a** were preserved, including covalent bonding to Cys491, hydrogen bonds in the hinge region, and the hydrogen bond with Asp644 (Figure 2D,E). Importantly, in the FGFR2^V564F^ model, the benzo[*d*]thiazole moiety of **8a** avoids steric clash with Phe564 and forms a π–π interaction with Phe645 (Figure 2E). Furthermore, docking of **8a** with FGFR3 revealed a similar binding mode to that of FGFR2 (Figure 2F). These computational findings supported the subsequent synthesis and biological evaluation of compound **8a**.

### Chemistry.

The synthetic routes for compounds **8a**–**g** are depicted in Scheme 1. The synthesis commenced with 4,6-dichloro-5-iodopyrimidine (**9**), which underwent nucleophilic aromatic substitution with aliphatic amines to provide intermediates **10a**-**b**. Subsequent Sonogashira coupling of **10a**-**b** with 3-(*tert*-butyldimethylsilyloxy)-1-propyne afforded compounds **11a**-**b**. Intramolecular cyclization of **11a**-**b** then yielded intermediates **12a**-**b**. Removal of the *tert*-butyldimethylsilyl (TBS) group from **12a**-**b** using tetrabutylammonium fluoride (TBAF), followed by benzylic chlorination with p-toluenesulfonyl chloride and subsequent intramolecular cyclization, furnished compounds **15a**-**b**. Treatment of **15a**-**b** with aqueous ammonia at 120 °C gave **16a**-**b**. Subsequent removal of the *tert*-butyloxycarbonyl (Boc) group with trifluoroacetic acid, followed by acylation with acryloyl chloride, afforded compounds **17a**-**b**. Iodination of **17a**-**b** with *N*-iodosuccinimide (NIS) provided **18a**-**b**, which were then subjected to Sonogashira coupling with trimethylsilylacetylene to produce **19a**-**b**. Finally, deprotection of **19a**-**b** with TBAF removed the trimethylsilyl (TMS) protecting group, and subsequent Sonogashira coupling with iodinated benzoxazole derivatives delivered the target compounds **8a**–**g**.

The synthetic routes to compounds **8h**–**s** are depicted in Scheme 2. Starting from 2-bromo-4,6-difluorobenzo[*d*]thiazole, Suzuki coupling with cyclopropylboronic acid afforded compound **22**. Treatment of **22** with lithium diisopropylamide (LDA) followed by quenching with *N,N*-dimethylformamide (DMF) provided compound **23**, which was subsequently converted to intermediate **24** *via* reaction with dimethyl(1-diazo-2-oxopropyl)phosphonate. Intermediates **28a**-**b** were synthesized from 2,4-difluoro-3-iodoaniline. Cyclization with ammonium thiocyanate and bromine (Br_2_) furnished compound **26**, which was then treated with *tert*-butyl nitrite to give compound **27**. Subsequent ring-opening with hydrazine hydrate (N_2_H_4_·H_2_O), followed by cyclization with the appropriate anhydride, delivered intermediates **28a**-**b**. Intermediates **31a**–**e** were prepared starting from compound **26**. Sonogashira coupling with trimethylsilylacetylene yielded compound **29**, which underwent bromination using CuBr_2_ and *tert*-butyl nitrite to afford compound **30**. Further functionalization *via* Suzuki or Negishi cross-coupling provided the desired intermediates **31a**–**e**. Finally, Sonogashira coupling of intermediates **24**, **28a**–**b**, and **31a**–**e** with the corresponding intermediates furnished the target compounds **8h**–**s**.

### Structural Optimization.

The Ba/F3 cellular assay was employed as the primary readout due to its ability to provide a functional and physiologically relevant measure of compound efficacy. In contrast to biochemical kinase assays, which assess target binding in an artificial system, the Ba/F3 model evaluates cell viability driven by oncogenic FGFR signaling within a native cellular context. This system captures key determinants of drug activity, including cell permeability, ATP competition, and kinase conformational states, while also validating oncogene addiction—ensuring that FGFR inhibition directly translates to antiproliferative effects. Moreover, it enables rapid profiling of selectivity and activity against clinically relevant FGFR2/FGFR3 mutants, positioning it a robust translational tool in drug discovery.

To validate the model systems, we first assessed the antiproliferative activity of the positive compound TAS-120 using IL-3–independent Ba/F3 cells expressing FGFR1, FGFR2, FGFR3, and FGFR2^V564F^, alongside parental Ba/F3 cells. TAS-120 potently inhibited BaF3-FGFR1/2/3 with low nanomolar IC_50_ values, and suppressed BaF3-FGFR2^V564F^ with an IC_50_ value of 153 nM, consistent with reported data (Table 1).^36^ Upon evaluating compound **8a** in the same panel, we observed that it maintained potent inhibitory activity against Ba/F3-FGFR2 and Ba/F3-FGFR3 cells (IC_50_ = 7.8 nM and 21.2 nM, respectively), while showing markedly reduced activity against Ba/F3-FGFR1 (IC_50_ = 337 nM) and parent Ba/F3 cells (IC_50_ >1000 nM). Notably, compound **8a** exhibited slightly greater potency than TAS-120 against BaF3-FGFR2^V564F^ cells. These findings support further modification of this novel scaffold.

We first investigated whether benzo[*d*]thiazole was the optimal substituent at the R^1^ position by designing compounds **8b**-**e,** each incorporating a hydrogen-bond-accepting nitrogen atom (Table 1). All these analogs displayed reduced potency relative to **8a** in inhibiting Ba/F3-FGFR2, Ba/F3-FGFR3, and BaF3-FGFR2^V564F^ cell growth. Inspired by the discovery of the pan-FGFR inhibitor KIN-3248, where fluorine atoms adjacent to the alkyne were shown to enhance activity against both wild-type and mutated FGFR kinases,^35^ we introduced a difluoro motif into compounds **8a** and **8c** to afford **8f** and **8g**, respectively. Evaluation of these difluoro-substituted analogs in Ba/F3 cells revealed enhanced inhibitory activity compared with their non-difluorinated counterparts for FGFR3 and FGFR2^V564F^ backgrounds. For instance, compound **8f** achieved IC_50_ values of 6.8 nM and 29.1 nM against Ba/F3-FGFR3 and Ba/F3-FGFR2^V564F^ cells, respectively—representing approximately threefold improvements over **8a**.

To guide further optimization, we performed covalent docking studies of compound **8f** bound to FGFR2. The model indicated that the benzo[*d*]thiazole moiety extends into the hydrophobic back pocket, where residual space may accommodate a small hydrophobic group (Figure 3). Accordingly, we designed compounds **8h–m** (Table 1). Among these, the methyl-substituted analog **8h** displayed enhanced potency against Ba/F3-FGFR3 cells while retaining activity against Ba/F3-FGFR2. In contrast, the ethyl- and cyclopropyl-substituted analogs **8i** and **8k** exhibited modestly reduced antiproliferative activity across Ba/F3-FGFR2, Ba/F3-FGFR3, and Ba/F3-FGFR2^V564F^ cells relative to **8f**, likely due to increased steric bulk. This trend of diminishing activity with larger substituents was consistently observed, with the weakest effects noted for the isopropyl (**8j)**, cyclobutyl (**8l)**, and cyclopentyl (**8m**) analogs.

To further improve the inhibitory activity, we re-examined the predicted binding model of **8f.** The model revealed that the piperidinyl ring is positioned near the P-loop, leaving a relatively large unoccupied space on the opposite side (Figure 3). We therefore sought to introduce a chiral methyl group with defined stereochemistry at this position. Specifically, a chiral methyl substituent was installed in the most potent analogs identified above (**8f**, **8h**, **8i**, and **8k**), yielding compounds **8n**, **8o**, **8r**, and **8s** (**KNT-0919**), respectively. In parallel, we designed compounds **8p** and **8q**, incorporating difluoromethyl and trifluoromethyl groups, respectively, given their superior metabolic stability relative to the methyl group in **8h** (Table 2). Introduction of the chiral methyl substituent resulted in a pronounced enhancement of antiproliferative potency across Ba/F3-FGFR1/2/3 and Ba/F3-FGFR2^V564F^ cells compared with the corresponding non-methylated analogs. For example, compound **8n**, derived from **8f** by appending a chiral methyl adjacent to the acrylamide warhead, exhibited markedly improved inhibitory activity against Ba/F3-FGFR2, FGFR3, and FGFR2^V564F^ cells, with IC_50_ values of 0.78, 0.63, and 0.89 nM, respectively. Among this series, the cyclopropyl-substituted analog **KNT-0919** demonstrated the most balanced overall profile, with potent inhibition of Ba/F3-FGFR2 (IC_50_ = 1.1 nM), Ba/F3-FGFR3 (IC_50_ = 0.20 nM), and Ba/F3-FGFR2^V564F^ (IC_50_ = 5.6 nM), while also achieving the highest isoform selectivity within this series (16-fold for FGFR2 over FGFR1 and 85-fold for FGFR3 over FGFR1). This compound was therefore selected for further characterization. It is worth noting that the FGFR2 enzymatic inhibitory activities of the compounds show a similar trend to the antiproliferative activity of Ba/F3 FGFR2 at the cellular level (Table 1 and 2).

### KNT-0919 Selectively Inhibits FGFR2/3 Kinase Activity.

The kinase inhibitory activities of **KNT-0919** against FGFR1-4 were evaluated using ADP-Glo assays. As shown in Figure 4A, **KNT-0919** potently inhibited FGFR2 and FGFR3, with IC_50_ values of 0.8 nM and 0.3 nM, respectively. In contrast, it exhibited reduced activity against FGFR1 (IC_50_ = 2.2 nM) and FGFR4 (IC_50_ = 20.3 nM). To characterize covalent inhibitory kinetics, steady-state kinetic parameters against FGFR1 and FGFR2 were determined (Table S1). **KNT‑0919** exhibited a *K*_i_ value of 0.4 nM and a *k*_inact_ of 0.00405 s^−1^ toward FGFR2, yielding a *k*_inact_/*K*_i_ efficiency constant of 0.01053 nM^−1^·s^−1^. By contrast, the respective parameters for FGFR1 were 5.3 nM (*K*_i_), 0.00599 s^−1^ (*k*_inact_), and 0.00113 nM^−1^·s^−1^ (k_inact_/K_i_). These results reveal that the preferential selectivity of **KNT-0919** of FGFR2 over FGFR1 is predominantly governed by reversible binding affinity rather than covalent bond formation rate. To further assess its selectivity, **KNT-0919** was profiled against a panel of 416 protein kinases at a concentration of 100 nM (Figure 4B, C and Table S3). Outside the FGFR family, only one wild-type kinase—rearranged-during-transfection (RET), a validated target for RET-altered cancers^37^—showed greater than 90% inhibition. Notably, **KNT-0919** also demonstrated potent inhibitory activity against multiple clinically relevant FGFR2 and FGFR3 mutant variants (Figure 4B, C). These findings indicate that **KNT-0919** selectively inhibits FGFR2/3 kinase activity.

### KNT-0919 Covalently Binds to FGFR2 and FGFR3.

To validate the covalent binding mechanism, intact protein mass spectrometry (MS) was performed. Incubation of recombinant FGFR2 with excess **KNT-0919** resulted in a distinct intact mass shift, consistent with the formation of a covalent **KNT-0919**-FGFR2 complex (Figure 5A). Covalent modification at Cys491 was confirmed by higher-energy collision-induced dissociation (HCD) MS/MS, which identified a peptide unique to the modified sample with a mass corresponding to the **KNT-0919** adduct (Figure 5B). We further determined the X-ray cocrystal structures of the FGFR2 and FGFR3 kinases in complex with **KNT-0919**. In the **KNT-0919**–FGFR2 complex (PDB: 22ZR) (Figure 5C), the tricyclic tetrahydropyrazino–pyrrolo–pyrimidinylamino core is anchored in the hinge region *via* hydrogen bonds with Glu565 and Ala567. The benzo[*d*]thiazole moiety extends into the hydrophobic back pocket, while the acrylamide warhead irreversibly binds to Cys491 in the P-loop. Notably, the acrylamide carbonyl forms two additional hydrogen bonds with Arg630, potentially stabilizing the covalent binding conformation. Due to poor definition of the activation loop in the FGFR2 structure, a predicted hydrogen bond between the thiazole nitrogen and Asp644 could not be clearly resolved. In contrast, this interaction was unambiguously observed in the **KNT-0919**–FGFR3 structure (PDB: 22ZS), specifically between the thiazole nitrogen and Asp635 (Figure 5D). Overall, the binding mode of **KNT-0919** in FGFR3 is consistent with that in FGFR2: the core forms hydrogen bonds with Glu556 and Ala558 in the hinge region, the acrylamide warhead covalently modifies Cys482, and its carbonyl engages in a hydrogen bond with Arg621. These results confirm that **KNT-0919** covalently binds to both FGFR2 and FGFR3.

### KNT-0919 Selectively Suppresses FGFR2-Dependent Cancer Cell Proliferation and Downstream Signaling, and Induces Apoptosis.

The antiproliferative activity of **KNT-0919** was evaluated in four cancer cell lines harboring distinct FGFR alterations: A204 (FGFR1 amplification), SNU16 (FGFR2 amplification), KATO III (FGFR2 amplification), KMS-11 (FGFR3 Y373C and fusion), MDA-MB-453 (FGFR4-Y367C mutant) and HEK-293T (Human embryonic kidney cell) (Figure 6A). **KNT-0919** exhibited potent activity in FGFR2-dependent SNU16 and KATO III cells (IC_50_ = 6.2 nM and 6.3 nM, respectively) and FGFR3-dependent KMS-11 cells (IC_50_ = 13.5 nM), while showing substantially reduced potency against A204 cells (IC_50_ = 75.3 nM), MDA-MB-453 cells (IC_50_ = 1310 nM), and HEK-293T cells (IC_50_ > 1000 nM). In addition, **KNT-0919** retained strong inhibitory activity against Ba/F3 cells expressing FGFR2 mutants, with IC_50_ values of 9.6 nM for FGFR2^V564I^ and 20.4 nM for FGFR2^N549K^. The effects of **KNT-0919** on FGFR2-mediated downstream signaling were assessed in SNU-16 cells by western blotting. As shown in Figure 6B, **KNT-0919** treatment led to dose-dependent inhibition of FGFR2 phosphorylation, as well as phosphorylation of downstream effectors fibroblast growth factor receptor substrate 2 α (FRS2α), Akt, and extracellular signal-regulated kinases 1 and 2 (ERK1/2). Moreover, **KNT-0919** induced apoptosis in SNU-16 cells in a dose-dependent manner, with approximately 28% apoptotic cells observed after 48 h of treatment at 100 nM. In contrast, under the same conditions, minimal apoptosis was observed in A204 cells (Figure 6C). These findings demonstrate that **KNT-0919** selectively inhibits FGFR2-dependent cancer cell proliferation and downstream signaling, and promotes apoptosis.

### KNT-0919 Exhibits Favorable Oral Bioavailability in Rats and Potent Antitumor Efficacy *In Vivo*.

The pharmacokinetic (PK) profile of **KNT-0919** was assessed in Sprague–Dawley (SD) rats following intravenous (1 mg/kg) and oral (10 mg/kg) administration. As summarized in Table 3, **KNT-0919** showed an oral bioavailability of 55.6%, with a maximum plasma concentration (C_max_) of 500 ng/mL and an exposure (AUC_0–∞_) of 1708 ng*h/mL. The *in vivo* efficacy was evaluated in an SNU-16 xenograft mouse model. **KNT-0919** was administrated orally at doses of 3, 10 and 30 mg/kg twice daily for 35 consecutive days. As shown in Figure 7A–C, **KNT-0919** suppressed tumor growth in a dose-dependent manner, with tumor growth inhibition (TGI) values of 78.6%, 97.4%, and 115.6% at 3, 10, and 30 mg/kg, respectively. Tumor regression was observed at the highest dose. All treatments were well tolerated, with no significant body weight loss during the study period (Figure 7D). *In vivo* pharmacodynamic assessment confirmed that **KNT-0919** treatment led to dose-dependent inhibition of phosphorylation of FGFR2 and its downstream signaling (Figure 7E). Analysis of tumor samples collected at the end of treatment revealed increased apoptosis in the treatment groups, as evidenced by H&E and TUNEL-staining (Figure 7F). We further conducted an efficacy study by including TAS-120 as a positive control. TAS-120 was administered orally once daily at 10 mg/kg, while **KNT-0919** was given orally at 10 mg/kg either once daily or twice daily for 22 consecutive days. As shown in Figure 7G, **KNT-0919** exhibited antitumor activity comparable to that of TAS-120 without significant body weight loss (Supporting Information Figure S1).

## CONCLUSIONS

Selective targeting of FGFR2 and/or FGFR3 has emerged as a promising therapeutic strategy for cancers driven by these specific isoforms; however, no such inhibitors have yet been approved for clinical use. Thus, there is a need for novel selective FGFR2/3 inhibitors with distinct chemical scaffolds. Here, we report the design and synthesis of a novel series of tricyclic tetrahydropyrazino-pyrrolo-pyrimidinylamino derivatives as irreversible FGFR2/3 inhibitors. Among these, the representative compound **KNT-0919** exhibited potent antiproliferative activity against Ba/F3 cells driven by FGFR2 and FGFR3, with IC_50_ values of 1.1 nM and 0.20 nM, respectively, and retained activity against Ba/F3 cells expressing FGFR2 gatekeeper mutants. In contrast, it showed reduced potency against Ba/F3-FGFR1 cells (IC_50_ = 17.1 nM) and parental Ba/F3 cells (IC_50_ > 1000 nM). In a panel of 416 kinases, **KNT-0919** selectively inhibited FGFR2 and FGFR3, including clinically relevant mutant variants. To better mitigate FGFR1-related systemic toxicities, the selectivity of this compound might be further improved. It is worth noting that we adopted a more rigid tricyclic scaffold and successfully enhanced the subtype selectivity toward FGFR2/3, further demonstrating conformation constraint is a useful strategy to improve the selectivity of kinase inhibitors.

Covalent binding of **KNT-0919** to FGFR2 and FGFR3 was confirmed by intact mass spectrometry and X-ray co-crystal structures. Furthermore, **KNT-0919** displayed favorable pharmacokinetic properties, including an oral bioavailability of 56%. Importantly, oral administration of **KNT-0919** led to significant tumor regression in an SNU-16 xenograft mouse model without observable toxicity. While more characterizations are needed to investigate its efficacy and safety profiles, these findings establish **KNT-0919** as a promising covalent FGFR2/3 inhibitor and support its further development for the treatment of FGFR2/3-driven malignancies, including those associated with acquired resistance mutations.

## EXPERIMENTAL SECTION

### General Methods for Chemistry.

All reagents and solvents in chemical synthesis were purchased from commercial vendors and used without further purification, unless indicated otherwise. All reactions were monitored by thin-layer chromatography (TLC) and visualized by UV light visualization (254 and 365 nm). All compounds were purified by a column chromatography on silica gel (200 - 300 mesh). The ^1^H, ^19^F and ^13^C NMR spectra were recorded on Agilent DD2 500 spectrometer (Agilent Technologies Inc., USA) or Bruker AVANCE 400/600 spectrometer (Bruker Company, Germany) and referenced with respect to appropriate internal standards or residual solvent peaks (CDCl_3_ = 7.26 ppm, DMSO-*d*_6_ = 2.50 ppm). The following abbreviations were used in reporting spectra, s (singlet), d (doublet), t (triplet), q (quartet), m (multiplet). The spectra of high-resolution mass (HRMS) were monitored by Bruker MaXis 4G TOF Mass Spectrometer and an ESI source. The purities of all final compounds were monitored by HPLC analysis with the Agilent 1200 system. HPLC condition: Triart C18 reversed-phase column, 5 *μ*m, 4.6 mm × 250 mm, and flow rate 1.0 mL/min, starting with a 15 min gradient from 0.1% TFA in water and acetonitrile 9:1 mixture to 0.1% TFA in acetonitrile, then ending with 0.1% TFA in acetonitrile for 5 min. The purity of all final compounds was confirmed to be > 95% by HPLC analysis with the Agilent 1260 system.

#### tert-butyl (2-((6-chloro-5-iodopyrimidin-4-yl)amino)ethyl)carbamate (**10a**).

4,6-dichloro-5-iodopyrimidine (8.25 g, 30.0 mmol) was dissolved in anhydrous acetonitrile (100 mL) in a 250 mL round-bottom flask. *N,N*-Diisopropylethylamine (DIPEA, 7.8 g, 60.0 mmol) and *tert*-butyl (2-aminoethyl)carbamate (5.70 g, 35.0 mmol) were then added sequentially, and the resulting mixture was stirred at room temperature for 14 h. The reaction was diluted with water and extracted with ethyl acetate. The combine organic layer was washed with brine, dried over anhydrous sodium sulfate and concentrated to afford the crude product **10a** as a white solid (10.5 g, 88%), which was used directly in the next step without further purification. ^1^H NMR (500 MHz, Chloroform-*d*) δ 8.22 (s, 1H), 6.31 (s, 1H), 4.87 (s, 1H), 3.61 – 3.54 (m, 2H), 3.46 – 3.39 (m, 2H), 1.44 (s, 9H). MS(ESI)m/z 398.0 [M+H]^+^.

#### tert-butyl (R)-(1-((6-chloro-5-iodopyrimidin-4-yl)amino)propan-2-yl)carbamate (**10b**).

Compound **10b** was prepared by following a similar procedure to that of **10a**. Yield, 92%. ^1^H NMR (400 MHz, Chloroform-*d*) δ 8.22 (s, 1H), 6.45 (s, 1H), 4.61 (s, 1H), 4.07 – 3.92 (m, 1H), 3.63 – 3.51 (m, 1H), 3.48 – 3.34 (m, 1H), 1.44 (s, 9H), 1.23 (d, *J* = 6.8 Hz, 3H). MS(ESI)m/z 412.0 [M+H]^+^.

#### tert-butyl (2-((5-(3-((tert-butyldimethylsilyl)oxy)prop-1-yn-1-yl)-6-chloropyrimidin-4-yl)amino)ethyl)carbamate (**11a**).

Compound **10a** (10.5 g, 26.4 mmol) was dissolved in anhydrous tetrahydrofuran (THF, 100 mL) in a 250 mL round-bottom flask. To this solution were added *tert*-butyldimethyl (prop-2-yn-1-yloxy)silane (7.40 g, 43.6 mmol), Pd(PPh_3_)_2_Cl_2_ (1.53 g, 2.18 mmol), CuI (0.83 g, 4.36 mmol), and Et_3_N (11.0 g, 109 mmol). The reaction mixture was stirred at 70 °C for 14 h under a nitrogen atmosphere. The reaction was cooled to room temperature and diluted with water and extracted with ethyl acetate. The organic layer was washed with brine, dried over anhydrous sodium sulfate, filtered, and concentrated. The crude product was purified by silica gel column chromatography (EA/PE = 30%) to afford compound **11a** (8.8 g, 76%). ^1^H NMR (500 MHz, Chloroform-*d*) δ 8.27 (s, 1H), 6.38 (s, 1H), 4.93 (s, 1H), 4.64 (s, 2H), 3.64 – 3.55 (m, 2H), 3.45 – 3.32 (m, 2H), 1.42 (s, 9H), 0.92 (s, 9H), 0.16 (s, 6H). MS(ESI)m/z 441 .2 [M+H]^+^.

#### tert-butyl (R)-(1-((5-(3-((tert-butyldimethylsilyl)oxy)prop-1-yn-1-yl)-6-chloropyrimidin-4-yl)amino)propan-2-yl)carbamate (**11b**).

Compound **11b** was prepared by following a similar procedure to that of **11a**. Yield, 83%. ^1^H NMR (400 MHz, Chloroform-*d*) δ 8.28 (s, 1H), 6.56 (s, 1H), 4.71 – 4.60 (m, 3H), 3.94 (s, 1H), 3.60 – 3.42 (m, 2H), 1.41 (s, 9H), 1.22 (d, *J* = 6.7 Hz, 3H), 0.94 (s, 9H), 0.18 (s, 6H). MS(ESI)m/z 454 .2 [M+H]^+^.

#### tert-butyl (2-(6-(((tert-butyldimethylsilyl)oxy)methyl)-4-chloro-7H-pyrrolo[2,3-d]pyrimidin-7-yl)ethyl)carbamate (**12a**).

Compound **11a** (8.80 g, 20.0 mmol) was dissolved in anhydrous acetonitrile (100 mL) in a 250 mL round-bottom flask. Cs_2_CO_3_ (9.75 g, 30.0 mmol) was added to the solution, and the resulting mixture was stirred at 90 °C for 6 h. The reaction was cooled to room temperature and diluted with water and extracted with ethyl acetate. The combine organic layer was washed with brine, dried over anhydrous sodium sulfate and concentrated. The crude product was further purified by silica gel column chromatography (EA/PE = 100%) to afford compound **12a** (6.7 g, 76%). ^1^H NMR (500 MHz, Chloroform-*d*) δ 8.60 (s, 1H), 6.49 (s, 1H), 4.92 (s, 1H), 4.86 (s, 2H), 4.46 (t, *J* = 5.9 Hz, 2H), 3.63 – 3.51 (m, 2H), 1.34 (s, 9H), 0.92 (s, 9H), 0.12 (s, 6H). MS (ESI) m/z 441.2 [M+H]^+^.

#### tert-butyl (R)-(1-(6-(((tert-butyldimethylsilyl)oxy)methyl)-4-chloro-7H-pyrrolo[2,3-d]pyrimidin-7-yl)propan-2-yl)carbamate (**12b**).

Compound **12b** was prepared by following a similar procedure to that of **12a**. Yield, 85%. ^1^H NMR (400 MHz, Chloroform-*d*) δ 8.61 (s, 1H), 6.48 (s, 1H), 5.00 – 4.84 (m, 3H), 4.36 (d, *J* = 7.1 Hz, 2H), 4.25 – 4.14 (m, 1H), 1.23 (d, *J* = 6.6 Hz, 3H), 1.17 (s, 9H), 0.93 (s, 9H), 0.14 (d, *J* = 9.9 Hz, 6H). MS (ESI) m/z 441.2[M+H]^+^. MS(ESI)m/z 454 .2 [M+H]^+^.

#### tert-butyl (2-(4-chloro-6-(hydroxymethyl)-7H-pyrrolo[2,3-d]pyrimidin-7-yl)ethyl)carbamate (**13a**).

Compound **12a** (8.80 g, 20.7 mmol) was dissolved in anhydrous tetrahydrofuran (THF, 100 mL) in a 250 mL round-bottom flask. Tetrabutylammonium fluoride (30.0 mL, 30.0 mmol, 1.0 M in THF) was added, and the resulting mixture was stirred at room temperature for 1 h. The reaction mixture was diluted with water and extracted with ethyl acetate. The combine organic layer was washed with brine, dried over anhydrous sodium sulfate and concentrated. The crude product was used directly in the next step without further purification.

#### tert-butyl (R)-(1-(4-chloro-6-(hydroxymethyl)-7H-pyrrolo[2,3-d]pyrimidin-7-yl)propan-2-yl)carbamate (**13b**).

Compound **13b** was prepared by following a similar procedure to that of **13a**.

#### tert-butyl (2-(4-chloro-6-(chloromethyl)-7H-pyrrolo[2,3-d]pyrimidin-7-yl)ethyl)carbamate (**14a**).

Compound **13a** was dissolved in anhydrous dichloromethane (100 mL) in a 250 mL round-bottom flask. Et_3_N (5.20 g, 1.6 mmol) and p-toluenesulfonyl chloride (4.75 g, 25.0 mmol) were added sequentially, and the resulting mixture was stirred at room temperature for 1 h. The reaction mixture was diluted with water and extracted with ethyl acetate. The combined organic layers were concentrated under reduced pressure to give the crude product, which was further purified by silica gel column chromatography (EA/PE= 100%) to afford compound **14a** (4.5 g, 63% for two steps). ^1^H NMR (500 MHz, Chloroform-*d*) δ 8.62 (s, 1H), 6.66 (s, 1H), 4.85 (s, 1H), 4.80 (s, 2H), 4.49 (t, *J* = 6.2 Hz, 2H), 3.63 – 3.54 (m, 2H), 1.34 (s, 9H). MS (ESI) m/z 344.1 [M+H]^+^.

#### tert-butyl (R)-(1-(4-chloro-6-(chloromethyl)-7H-pyrrolo[2,3-d]pyrimidin-7-yl)propan-2-yl)carbamate (**14b**).

Compound **14b** was prepared by following a similar procedure to that of **14a**. Yield, 79% for two steps. ^1^H NMR (400 MHz, Chloroform-*d*) δ 8.64 (s, 1H), 6.67 (s, 1H), 4.96 – 4.79 (m, 2H), 4.75 – 4.64 (m, 1H), 4.50 – 4.30 (m, 2H), 4.20 – 4.08 (m, 1H), 1.26 (d, *J* = 6.7 Hz, 3H), 1.19 (s, 9H). MS (ESI) m/z 358.1 [M+H]^+^.

#### tert-butyl 4-chloro-8,9-dihydropyrazino[1′,2′:1,5]pyrrolo[2,3-d]pyrimidine-7(6H)-carboxylate (**15a**).

Compound **14a** (4.50 g, 13.6 mmol) was dissolved in anhydrous acetonitrile (50 mL) in a 100 mL round-bottom flask. Cs_2_CO_3_ (6.50 g, 20.0 mmol) was added to the solution. The reaction mixture was stirred at 70 °C for 14 h under a nitrogen atmosphere. The reaction was cooled to room temperature and diluted with water and extracted with ethyl acetate. The combine organic layer was washed with brine, dried over anhydrous sodium sulfate and concentrated. The crude product was further purified by silica gel column chromatography (EA/PE= 100%) to afford compound **15a** (3.10 g, 71%). ^1^H NMR (500 MHz, Chloroform-*d*) δ 8.61 – 8.56 (m, 1H), 6.38 (s, 1H), 4.85 (s, 2H), 4.27 (t, *J* = 5.7 Hz, 2H), 3.92 (t, *J* = 5.9 Hz, 2H), 1.51 (s, 9H). MS (ESI) m/z 309.1 [M+H]^+^.

#### tert-butyl (R)-4-chloro-8-methyl-8,9-dihydropyrazino[1′,2′:1,5]pyrrolo[2,3-d]pyrimidine-7(6H)-carboxylate (**15b**).

Compound **15b** was prepared by following a similar procedure to that of **15a**. Yield, 73%. ^1^H NMR (400 MHz, Chloroform-*d*) δ 8.60 (s, 1H), 6.43 (s, 1H), 5.07 (d, *J* = 17.7, 1.4 Hz, 1H), 4.97 – 4.70 (m, 1H), 4.56 (d, *J* = 17.7, 1.3 Hz, 1H), 4.43 (d, *J* = 12.7, 1.8 Hz, 1H), 4.17 – 4.10 (m, 1H), 1.52 (s, 9H), 1.15 (d, *J* = 6.9 Hz, 3H). MS (ESI) m/z 322.1 [M+H]^+^.

#### tert-butyl 4-amino-8,9-dihydropyrazino[1′,2′:1,5]pyrrolo[2,3-d]pyrimidine-7(6H)-carboxylate (**16a**).

Compound **15a** (3.1 g, 10.06 mmol) was dissolved in 1,4-dioxane (20 mL) in a 200 mL sealed tube. Aqueous ammonia (40 mL, 27% in H_2_O) was added, and the resulting mixture was stirred at 120 °C for 4 h. the reaction mixture was cooled to room temperature and concentrated under reduced pressure to give the crude product, which was further purified by silica gel column chromatography (MeOH/DCM = 10%) to afford compound **16a** (1.5 g, 49%). ^1^H NMR (400 MHz, DMSO-*d*_6_) δ 8.02 (s, 1H), 6.95 (s, 2H), 6.33 (s, 1H), 4.68 (s, 2H), 4.04 (t, *J* = 6.4, 4.7 Hz, 2H), 3.78 (t, *J* = 5.6 Hz, 2H), 1.44 (s, 9H). MS (ESI) m/z 290.2 [M+H]^+^.

#### tert-butyl (R)-4-amino-8-methyl-8,9-dihydropyrazino[1′,2′:1,5]pyrrolo[2,3-d]pyrimidine-7(6H)-carboxylate (**16b**).

Compound **16b** was prepared by following a similar procedure to that of **16a**. Yield, 45%. ^1^H NMR (400 MHz, Chloroform-*d*) δ 8.30 (s, 1H), 6.16 (s, 1H), 5.26 (s, 2H), 4.99 – 4.90 (m, 1H), 4.87 – 4.59 (m, 1H), 4.51 (d, *J* = 17.0, 1.2 Hz, 1H), 4.37 (d, *J* = 12.8, 1.9 Hz, 1H), 4.10 – 4.03 (m, 1H), 1.52 (s, 9H), 1.12 (d, *J* = 6.8 Hz, 3H). MS (ESI) m/z 303.2 [M+H]^+^.

#### 1-(4-amino-8,9-dihydropyrazino[1′,2′:1,5]pyrrolo[2,3-d]pyrimidin-7(6H)-yl)prop-2-en-1-one (**17a**).

Compound **16a** (1.5 g, 5.18 mmol) was dissolved in dichloromethane (10 mL) in a 50 mL round-bottom flask. Trifluoroacetic acid (5 mL) was added dropwise at room temperature, and the mixture was stirred for 2 h. The reaction mixture was concentrated under reduced pressure to afford yellow oil, which was used directly in the next step without further purification. The above crude product was dissolved in anhydrous tetrahydrofuran (THF, 50 mL) in a 100 mL round-bottom flask. Acrylic acid (373.0 mg, 5.18 mmol), Et_3_N (1.57 g, 15.54 mmol) and T3P (4.94 g, 7.77 mmol, 50% in EtOAc) were added sequentially. The resulting mixture was stirred at room temperature for 1 h. The reaction was diluted with water and extracted with ethyl acetate. The combined organic layers were concentrated under reduced pressure to give the crude product, which was further purified by silica gel column chromatography (MeOH/DCM = 10%) to afford compound **17a** (855 mg, 57% yield for two steps) as a white solid. ^1^H NMR (400 MHz, DMSO-*d*_6_) δ 8.04 (s, 1H), 6.99 (s, 2H), 6.38 (s, 1H), 6.19 (d, *J* = 16.7 Hz, 1H), 5.80 – 5.74 (m, 1H), 5.05 – 4.78 (m, 2H), 4.13 – 3.97 (m, 4H). MS (ESI) m/z 244.1 [M+H]^+^.

#### (R)-1-(4-amino-8-methyl-8,9-dihydropyrazino[1′,2′:1,5]pyrrolo[2,3-d]pyrimidin-7(6H)-yl)prop-2-en-1-one (**17b**).

Compound **17b** was prepared by following a similar procedure to that of **17a**. Yield, 36%. ^1^H NMR (400 MHz, Chloroform-*d*) δ 8.08 (s, 1H), 6.85 (s, 1H), 6.69 – 6.58 (m, 1H), 6.41 (dd, *J* = 16.7, 1.8 Hz, 1H), 5.84 (dd, *J* = 10.4, 1.8 Hz, 1H), 5.27 (s, 2H), 4.86 – 4.63 (m, 2H), 4.63 – 4.40 (m, 2H), 4.18 – 4.07 (m, 1H), 1.20 (d, *J* = 6.9 Hz, 3H). MS (ESI) m/z 257.1 [M+H]^+^.

#### 1-(4-amino-5-iodo-8,9-dihydropyrazino[1′,2′:1,5]pyrrolo[2,3-d]pyrimidin-7(6H)-yl)prop-2-en-1-one (**18a**).

Compound **17a** (855.0 mg, 3.52 mmol) was dissolved in tetrahydrofuran (THF, 15 mL) in a 50 mL round-bottom flask. NIS (791 mg, 3.52 mmol) was added, and the resulting mixture was stirred at room temperature for 4 h. Saturated aqueous sodium thiosulfate solution (30 mL) was added to quench the reaction. The mixture was extracted with ethyl acetate. The combined organic layers were concentrated under reduced pressure to give the crude product, which was further purified by silica gel column chromatography (MeOH/DCM = 7%) to afford compound **18a** (1.03g, 79% yield) as a white solid. ^1^H NMR (500 MHz, Chloroform-*d*) δ 8.25 (s, 1H), 6.70 – 6.60 (m, 1H), 6.41 (dd, *J* = 16.7 Hz, 1H), 5.94 – 5.62 (m, 3H), 4.86 – 4.70 (m, 2H), 4.28 – 4.18 (m, 2H), 4.17 – 3.94 (m, 2H). MS (ESI) m/z 369.0 [M+H]^+^.

#### (R)-1-(4-amino-5-iodo-8-methyl-8,9-dihydropyrazino[1′,2′:1,5]pyrrolo[2,3-d]pyrimidin-7(6H)-yl)prop-2-en-1-one (**18b**).

Compound **18b** was prepared by following a similar procedure to that of **18a**. Yield, 65% .^1^H NMR (400 MHz, Chloroform-*d*) δ 8.17 (s, 1H), 6.71 – 6.61 (m, 1H), 6.44 (dd, *J* = 16.7, 1.7 Hz, 1H), 5.87 (dd, *J* = 10.6, 1.7 Hz, 1H), 5.38 – 4.69 (m, 2H), 4.54 – 4.33 (m, 2H), 4.16 – 4.05 (m, 1H), 1.24 (d, *J* = 6.9 Hz, 3H). MS (ESI) m/z 383.0 [M+H]^+^.

#### 1-(4-amino-5-ethynyl-8,9-dihydropyrazino[1′,2′:1,5]pyrrolo[2,3-d]pyrimidin-7(6H)-yl)prop-2-en-1-one (**19a**).

Compound **18a** (1.03 g, 2.79 mmol) was dissolved in *N,N*-dimethylformamide (10 mL) in a 25 mL round-bottom flask. To this solution were added trimethylsilylacetylene (1.37 g, 13.95 mmol), Pd(PPh^3^)_2_Cl_2_ (197 mg, 0.28 mmol), CuI (106 mg, 0.56 mmol), and Et_3_N (2.82 g, 27.9 mmol). The reaction mixture was stirred at 70 °C for 12 h under a nitrogen atmosphere. The reaction was cooled to room temperature and diluted with water and extracted with ethyl acetate. The combined organic layers were concentrated under reduced pressure to give the crude product. The crude product was further purified by silica gel column chromatography (MeOH/DCM = 8%) to afford compound **19a** (673 mg, 71% yield) as a white solid. ^1^H NMR (400 MHz, Chloroform-*d*) δ 8.29 (s, 1H), 6.75 – 6.60 (m, 1H), 6.43 (dd, *J* = 16.7 Hz, 1H), 5.91 – 5.80 (m, 1H), 5.68 (s, 2H), 5.09 – 4.92 (m, 2H), 4.32 – 4.18 (m, 2H), 4.18 – 3.92 (m, 2H), 0.28 (s, 9H). MS (ESI) m/z 339.2 [M+H]^+^.

#### (R)-1-(4-amino-5-ethynyl-8-methyl-8,9-dihydropyrazino[1′,2′:1,5]pyrrolo[2,3-d]pyrimidin-7(6H)-yl)prop-2-en-1-one (**19b**).

Compound **19b** was prepared by following a similar procedure to that of **19a**. Yield, 68%.^1^H NMR (400 MHz, Chloroform-*d*) δ 8.27 (s, 1H), 6.71 – 6.62 (m, 1H), 6.44 (dd, *J* = 16.7, 1.8 Hz, 1H), 5.84 (dd, *J* = 10.6 Hz, 1H), 5.66 (s, 2H), 5.49 – 5.01 (m, 2H), 4.80 – 4.63 (m, 1H), 4.53 – 4.35 (m, 1H), 4.11 – 3.95 (m, 1H), 1.19 (d, *J* = 6.9 Hz, 3H), 0.29 (s, 9H). MS (ESI) m/z 353.2 [M+H]^+^.

#### 1-(4-amino-5-ethynyl-8,9-dihydropyrazino[1′,2′:1,5]pyrrolo[2,3-d]pyrimidin-7(6H)-yl)prop-2-en-1-one (**20a**).

compound **19a** (773 mg, 1.98 mmol) was dissolved in tetrabutylammonium fluoride (2.97 mL, 2.97 mmol, 1.0 M in THF) in a 25 mL round-bottom flask. The reaction mixture was stirred at room temperature for 2 h. The reaction mixture was diluted with water and extracted with ethyl acetate. The combined organic layers were washed with brine, dried over anhydrous sodium sulfate and concentrated to afford compound **20a**, which was used directly in the next step without further purification.

#### (R)-1-(4-amino-5-ethynyl-8-methyl-8,9-dihydropyrazino[1′,2′:1,5]pyrrolo[2,3-d]pyrimidin-7(6H)-yl)prop-2-en-1-one (**20b**).

Compound **20b** was prepared by following a similar procedure to that of **20a**, and was used directly in the next step without further purification.

#### 1-(4-amino-5-(benzo[d]thiazol-5-ylethynyl)-8,9-dihydropyrazino[1′,2′:1,5]pyrrolo[2,3-d]pyrimidin-7(6H)-yl)prop-2-en-1-one (**8a**).

Compound **20a** (60 mg, 0.22 mmol) was dissolved in *N,N*-dimethylformamide (2 mL). To this solution were added 5-iodobenzo[*d*]thiazole (70 mg, 0.27 mmol), Pd(PPh_3_)_2_Cl_2_ (15 mg, 0.022 mmol), CuI (8 mg, 0.044 mmol), and Et_3_N (111 mg, 1.1 mmol). The reaction mixture was stirred at 70 °C for 6 h under a nitrogen atmosphere. The reaction was cooled to room temperature and diluted with water and extracted with ethyl acetate. The combined organic layers were concentrated under reduced pressure to give the crude product, which was further purified by silica gel column chromatography (MeOH/DCM = 8%) to afford compound **8a** (28.6 mg, 34% yield) as a yellow solid. ^1^H NMR (400 MHz, DMSO-*d*_6_) δ 9.48 (s, 1H), 8.49 – 8.36 (m, 1H), 8.23 (d, *J* = 8.3 Hz, 1H), 8.17 (s, 1H), 7.80 – 7.68 (m, 1H), 7.12 – 6.53 (m, 3H), 6.23 (d, *J* = 16.6 Hz, 1H), 5.81 (d, *J* = 10.4 Hz, 1H), 5.19 – 4.98 (m, 2H), 4.22 – 4.00 (m, 4H). ^13^C NMR (151 MHz, DMSO) δ 164.35, 157.03, 156.43, 152.52, 151.59, 148.31, 133.80, 133.04, 127.82, 127.48, 127.32, 124.78, 122.27, 120.14, 100.87, 92.86, 88.23, 81.48, 41.70, 40.62, 39.45. HRMS (ESI) for C_21_H_17_N_6_OS [M+H]^+^, calcd: 401.1179, found: 401.1179. HPLC purity: 95.67%.

#### 1-(4-amino-5-(benzo[d]oxazol-5-ylethynyl)-8,9-dihydropyrazino[1′,2′:1,5]pyrrolo[2,3-d]pyrimidin-7(6H)-yl)prop-2-en-1-one (**8b**).

Compound **8b** was prepared by following a similar procedure to that of **8a**. ^1^H NMR (400 MHz, DMSO-*d*_6_) δ 8.84 (s, 1H), 8.26 – 8.07 (m, 2H), 7.85 (d, *J* = 8.4 Hz, 1H), 7.79 – 7.67 (m, 1H), 7.14 – 6.52 (m, 3H), 6.23 (d, *J* = 16.2 Hz, 1H), 5.81 (d, *J* = 10.3 Hz, 1H), 5.15 – 4.94 (m, 2H), 4.23 – 4.01 (m, 4H). ^13^C NMR (151 MHz, DMSO) δ 165.43, 157.49, 155.77, 152.64, 149.64, 149.35, 140.43, 134.68, 129.54, 128.89, 128.39, 123.35, 119.82, 112.12, 101.97, 93.79, 89.33, 81.60, 42.78, 41.68, 40.53. HRMS (ESI) for C_21_H_17_N_6_O_2_ [M+H]^+^, calcd: 385.1408, found: 385.1408. HPLC purity: 95.82%.

#### 1-(4-amino-5-((1-methyl-1H-benzo[d]imidazol-5-yl)ethynyl)-8,9-dihydropyrazino[1′,2′:1,5]pyrrolo[2,3-d]pyrimidin-7(6H)-yl)prop-2-en-1-one (**8c**).

Compound **8c** was prepared by following a similar procedure to that of **8a**. ^1^H NMR (500 MHz, DMSO-*d*_6_) δ 8.29 (s, 1H), 8.12 (s, 1H), 8.05 – 7.85 (m, 1H), 7.63 (s, 1H), 7.57 – 7.42 (m, 1H), 7.16 – 6.33 (m, 3H), 6.20 (d, *J* = 16.6 Hz, 1H), 5.87 – 5.70 (m, 1H), 5.16 – 4.85 (m, 2H), 4.22 – 3.96 (m, 4H), 3.85 (s, 3H). ^13^C NMR (151 MHz, DMSO) δ 165.44, 157.53, 157.02, 152.61, 149.25, 146.55, 134.00, 132.06, 128.89, 128.41, 126.07, 122.74, 116.11, 111.24, 102.01, 95.16, 89.80, 80.28, 42.81, 41.66, 40.53, 31.35. HRMS (ESI) for C_22_H_20_N_7_O [M+H]^+^, calcd: 398.1724, found: 398.1716. HPLC purity: 98.43%.

#### 1-(4-amino-5-(pyrazolo[1,5-a]pyridin-6-ylethynyl)-8,9-dihydropyrazino[1′,2′:1,5]pyrrolo[2,3-d]pyrimidin-7(6H)-yl)prop-2-en-1-one (**8d**).

Compound **8d** was prepared by following a similar procedure to that of **8a**.^1^H NMR (600 MHz, DMSO-*d*_6_) δ 9.28 – 9.17 (m, 1H), 8.36 – 8.01 (m, 2H), 7.74 (d, *J* = 9.1 Hz, 1H), 7.48 – 7.31 (m, 1H), 7.12 – 6.94 (m, 1H), 6.95 – 6.37 (m, 3H), 6.22 (d, *J* = 16.2 Hz, 1H), 5.80 (d, *J* = 10.9 Hz, 1H), 5.23 – 4.88 (m, 2H), 4.22 – 4.02 (m, 4H). ^13^C NMR (151 MHz, DMSO) δ 164.34, 156.46, 151.53, 148.28, 142.34, 137.91, 133.86, 133.35, 130.74, 127.80, 127.33, 125.23, 117.38, 107.32, 97.19, 89.60, 88.20, 81.89, 41.69, 40.62, 39.46. HRMS (ESI) for C_21_H_18_N_7_O [M+H]^+^, calcd: 384.1567, found: 384.1560. HPLC purity: 96.98%.

#### 1-(4-amino-5-(imidazo[1,2-a]pyridin-6-ylethynyl)-8,9-dihydropyrazino[1′,2′:1,5]pyrrolo[2,3-d]pyrimidin-7(6H)-yl)prop-2-en-1-one (**8e**).

Compound **8e** was prepared by following a similar procedure to that of **8a**. ^1^H NMR (500 MHz, DMSO-*d*_6_) δ 8.55 (s, 1H), 8.12 (s, 1H), 8.02 – 7.86 (m, 2H), 7.63 (s, 1H), 7.12 – 6.57 (m, 4H), 6.29 – 6.12 (m, 1H), 5.88 – 5.70 (m, 1H), 5.23 – 4.80 (m, 2H), 4.15 – 3.99 (m, 4H). ^13^C NMR (151 MHz, DMSO) δ 169.73, 164.35, 156.39, 151.62, 148.39, 134.23, 133.75, 127.82, 127.56, 127.32, 126.65, 118.72, 117.68, 113.54, 100.72, 92.26, 88.07, 83.31, 41.67, 40.65, 39.45. HRMS (ESI) for C_21_H_18_N_7_O [M+H]^+^, calcd: 384.1567, found: 384.1567. HPLC purity: 98.09%.

#### 1-(4-amino-5-((4,6-difluoro-1-methyl-1H-benzo[d]imidazol-5-yl)ethynyl)-8,9-dihydropyrazino[1′,2′:1,5]pyrrolo[2,3-d]pyrimidin-7(6H)-yl)prop-2-en-1-one (**8f**).

Compound **8f** was prepared by following a similar procedure to that of **8a**. ^1^H NMR (400 MHz, DMSO-*d*_6_) δ 8.35 (s, 1H), 8.18 (s, 1H), 7.62 (d, *J* = 9.1 Hz, 1H), 7.39 – 6.31 (m, 3H), 6.23 (d, *J* = 16.2 Hz, 1H), 5.81 (d, *J* = 10.5, 2.2 Hz, 1H), 5.15 – 4.93 (m, 2H), 4.23 – 3.99 (m, 4H), 3.86 (s, 3H). ^13^C NMR (151 MHz, DMSO) δ 164.36, 156.69, 156.48, 151.77, 151.73, 148.30, 146.41, 135.80, 133.46, 127.85, 127.64, 127.27, 101.14, 93.95, 93.76, 89.78, 87.89, 81.04, 41.64, 40.62, 39.46, 30.72. HRMS (ESI) for C_22_H_18_F_2_N_7_O [M+H]^+^, calcd: 434.1535, found: 434.1534. HPLC purity: 97.43%.

#### 1-(4-amino-5-((4,6-difluorobenzo[d]thiazol-5-yl)ethynyl)-8,9-dihydropyrazino[1′,2′:1,5]pyrrolo[2,3-d]pyrimidin-7(6H)-yl)prop-2-en-1-one (**8g**).

Compound **8g** was prepared from intermediate **27** by following a similar procedure to that of **8a**. ^1^H NMR (500 MHz, DMSO-*d*_6_) δ 9.44 (s, 1H), 8.16 – 8.09 (m, 2H), 7.34 – 6.23 (m, 3H), 6.20 (d, *J* = 16.3 Hz, 1H), 5.79 (d, *J* = 10.3 Hz, 1H), 5.11 – 4.97 (m, 2H), 4.17 – 4.01 (m, 4H). ^13^C NMR (151 MHz, DMSO) δ 164.38, 158.03, 157.69, 156.40, 153.76, 151.85, 148.42, 138.10, 135.70, 134.37, 127.89, 127.25, 104.60, 100.93, 99.10, 91.82, 87.35, 80.28, 41.59, 40.66, 39.46. HRMS (ESI) for C_21_H_15_F_2_N_6_OS [M+H]^+^, calcd: 437.0991, found: 437.0983. HPLC purity: 95.74%.

#### 2-cyclopropyl-4,6-difluorobenzo[d]thiazole (**22**).

2-bromo-4,6-difluorobenzo[*d*]thiazole (5.60 g, 22.4 mmol) was dissolved in 1,4-dioxane (50 mL), followed by the addition of cyclopropylboronic acid (6.00 g, 67.2 mmol), potassium phosphate (14.2 g, 67.2 mmol), and Pd(dppf)Cl_2_ (1.60 g, 2.24 mmol). The reaction mixture was stirred at 100 °C for 14 h under a nitrogen atmosphere. The mixture was extracted with ethyl acetate. The combined organic layers were concentrated under reduced pressure to afford a crude product, which was purified by silica gel column chromatography (EA/PE = 20%) to give compound **22** (3.80 g, 80% yield) as a white solid. ^1^H NMR (500 MHz, Chloroform-*d*) δ 7.28 (d, *J* = 8.0, 1.8 Hz, 1H), 6.93 (t, *J* = 9.6, 2.1 Hz, 1H), 2.44 – 2.36 (m, 1H), 1.28 – 1.20 (m, 4H). MS (ESI) m/z 236.0[M+H]^+^.

#### 2-cyclopropyl-4,6-difluorobenzo[d]thiazole-5-carbaldehyde (**23**).

Compound **22** (3.80 g, 18.0 mmol) was dissolved in THF (50 mL), and the resulting solution was cooled to −78 °C under a nitrogen atmosphere. A solution of LDA (21.6 mL, 21.6 mmol, 1.0 M in THF) was then added dropwise, and the mixture was stirred at this temperature for 1 h. *N,N*-Dimethylformamide (2.00 g, 25.0 mmol) was added, and the reaction was continued to stir at −78 °C for an additional 2 h. The reaction mixture was quenched with saturated aqueous ammonium chloride (50 mL). The mixture was extracted with ethyl acetate and the combined organic layers were concentrated under reduced pressure to afford a crude product, which was purified by silica gel column chromatography (EA/PE = 50%) to give compound **23** (3.20 g, 71% yield) as a white solid. ^1^H NMR (500 MHz, Chloroform-*d*) δ 10.45 (s, 1H), 7.38 (d, *J* = 9.4, 1.5 Hz, 1H), 2.46 – 2.38 (m, 1H), 1.32 – 1.28 (m, 4H). MS (ESI) m/z 239.0 [M+H]^+^.

#### 2-cyclopropyl-5-ethynyl-4,6-difluorobenzo[d]thiazole (**24**).

Compound **23** (3.20 g, 12.8 mmol) was dissolved in methanol (30 mL). K_2_CO_3_ (2.76 g, 20.0 mmol) and dimethyl (1-diazo-2-oxopropyl)phosphonate (2.88 g, 15.0 mmol) were added to the solution, and the mixture was stirred at room temperature for 12 h. The reaction mixture was concentrated under reduced pressure to obtain a crude product, which was purified by silica gel column chromatography (EA/PE = 20%) to give compound **24** (2.2 g, 73% yield) as a white solid. ^1^H NMR (500 MHz, Chloroform-*d*) δ 7.32 (d, *J* = 8.0, 1.5 Hz, 1H), 3.56 (s, 1H), 2.43 – 2.36 (m, 1H), 1.29 – 1.23 (m, 4H). MS (ESI) m/z 235.0 [M+H]^+^.

#### 4,6-difluoro-5-iodobenzo[d]thiazol-2-amine (**26**).

2,4-difluoro-3-iodoaniline (3 g, 11.8 mmol) and ammonium thiocyanate (3.58 g, 47 mmol) were dissolved in AcOH(50 ml). To the suspension solution was added dropwise Br_2_ (3.78 g, 23.6 mmol) at 0 °C. The reaction mixture was stirred at room temperature overnight. The mixture was poured into ice aqueous ammonia and extracted with ethyl acetate. The combined organic layers were washed with brine, dried over anhydrous sodium sulfate and concentrated. The crude product was further purified by silica gel column chromatography (EA/PE = 80%) to afford compound **26** (2.3 g, 62% yield) as a yellow solid. ^1^H NMR (500 MHz, DMSO-*d*_6_) δ 7.79 (s, 2H), 7.55 (d, *J* = 6.0 Hz, 1H). MS (ESI) m/z 312.9 [M+H]^+^.

#### 4,6-difluoro-5-iodobenzo[d]thiazole (**27**).

Compound **26** (1.0 g, 3.2 mmol) was dissolved in 1,4-dioxane (10 mL) in a 25 mL round-bottom flask. To this solution were added dropwise *tert*-butyl nitrite (495 mg, 4.8 mmol). The reaction mixture was stirred at 85 °C for 6 h. The reaction was cooled to room temperature and diluted with water and extracted with ethyl acetate. The combined organic layers were concentrated under reduced pressure to give the crude product, which was further purified by silica gel column chromatography (EA/PE = 2%) to afford compound **27** (703 mg, 74% yield) as a white solid. ^1^H NMR (500 MHz, DMSO-*d*_6_) δ 9.40 (s, 1H), 8.04 (d, 1H). MS (ESI) m/z 297.9 [M+H]^+^.

#### 2-(difluoromethyl)-4,6-difluoro-5-iodobenzo[d]thiazole (**28a**).

Compound **27** (100 g, 0.34 mmol) was dissolved in EtOH (2 ml). To this solution were added N_2_H_4_·H_2_O (471 mg, 50%). The reaction mixture was stirred at 80 °C for 6 h. The reaction was cooled to room temperature and concentrated under reduced pressure to give the crude product, which was used directly in the next step without further purification. The above crude product was dissolved in *N*-methylpyrrolidone(2 ml). To this solution were added dropwise difluoroacetic anhydride at 0 °C. The reaction mixture was stirred at 140 °C for 3 h. The reaction was cooled to room temperature and diluted with water and extracted with ethyl acetate. The combined organic layers were concentrated under reduced pressure to give the crude product, which was further purified by silica gel column chromatography (EA/PE = 2%) to afford compound **28a** (42 mg, 36% yield) as a white solid. ^1^H NMR (500 MHz, DMSO-*d*_6_) δ 8.12 (d, *J* = 7.5, 1.6 Hz, 1H), 7.50 (t, *J* = 53.6, 2.9 Hz, 1H). MS (ESI) m/z 347.9 [M+H]^+^.

#### 4,6-difluoro-5-iodo-2-(trifluoromethyl)benzo[d]thiazole (**28b**).

Compound **28b** was prepared by following a similar procedure to that of **28a**. Yield, 14%.^1^H NMR (500 MHz, Chloroform-*d*) δ 7.53 (d, *J* = 6.7, 2.0 Hz, 1H). MS (ESI) m/z 365.9 [M+H]^+^.

#### 4,6-difluoro-5-((trimethylsilyl)ethynyl)benzo[d]thiazol-2-amine (**29**).

Compound **26** (2 g, 6.4 mol) was dissolved in *N,N*-dimethylformamide (30 mL). To this solution was added trimethylsilylacetylene (32 mg, 3.1 mmol), Pd(PPh_3_)_2_Cl_2_ (420 mg, 0.6 mmol), CuI (243 mg, 1.2 mmol), and Et_3_N (3.2 g, 32 mmol). The reaction mixture was stirred at 70 °C for 6 h under a nitrogen atmosphere. The reaction was cooled to room temperature and diluted with water and extracted with ethyl acetate. The combined organic layers were concentrated under reduced pressure to give the crude product, which was further purified by silica gel column chromatography (EA/PE = 80%) to afford compound **29** (1.17 g, 65% yield) as a yellow solid. ^1^H NMR (500 MHz, Chloroform-*d*) δ 7.10 (d, *J* = 7.9, 1.6 Hz, 1H), 6.28 (s, 2H), 0.28 (s, 9H). MS (ESI) m/z 283.1 [M+H]^+^.

#### 2-bromo-4,6-difluoro-5-((trimethylsilyl)ethynyl)benzo[d]thiazole (**30**).

Compound **29** (1.35 g, 4.8 mmol) and CuBr_2_ (1.39 g, 6.2 mmol) was dissolved in CH_3_CN (20 mL) in a 50 mL round-bottom flask. To this solution was added dropwise *tert*-butyl nitrite (639 mg, 6.2 mmol). The reaction mixture was stirred at room temperature for 6 h. The reaction was diluted with water and extracted with ethyl acetate. The combined organic layers were concentrated under reduced pressure to give the crude product, which was further purified by silica gel column chromatography (EA/PE = 2%) to afford compound **30** (1.45 g, 87% yield) as a yellow solid. ^1^H NMR (500 MHz, Chloroform-*d*) δ 7.32 (d, 1H), 0.29 (s, 9H). MS (ESI) m/z 346.0 [M+H]^+^.

#### 4,6-difluoro-2-methyl-5-((trimethylsilyl)ethynyl)benzo[d]thiazole (**31a**).

Compound **30** (346 mg, 1.0 mol) was dissolved in 1,4-dioxane (5 mL). To this solution was added trimethylboroxine (375 mg, 3 mmol, 3.5M in THF), Pd(dppf)Cl_2_ (110 mg, 0.15 mmol) and K_3_PO_3_ (848 mg, 4 mmol). The reaction mixture was stirred at 100 °C for 6 h under a nitrogen atmosphere. The reaction was cooled to room temperature and concentrated under reduced pressure to give the crude product, which was further purified by silica gel column chromatography (EA/PE = 2%) to afford compound **31a** (183 mg, 65% yield) as a white solid. ^1^H NMR (400 MHz, Chloroform-*d*) δ 7.33 (d, *J* = 7.9, 1.5 Hz, 1H), 2.84 (s, 3H), 0.31 (s, 9H). MS (ESI) m/z 282.1 [M+H]^+^.

#### 2-ethyl-4,6-difluoro-5-((trimethylsilyl)ethynyl)benzo[d]thiazole (**31b**).

Compound **30** (346 mg, 1.0 mol) was dissolved in 1,4-dioxane (5 mL). To this solution was added diethylzinc (247 mg, 2.0 mmol, 1M in Hexanes) and Pd(dppf)Cl_2_ (73 mg, 0.1 mmol). The reaction mixture was stirred at 95 °C for 6 h under a nitrogen atmosphere. The reaction was cooled to room temperature and concentrated under reduced pressure to give the crude product, which was further purified by silica gel column chromatography (EA/PE = 2%) to afford compound **31b** (251mg, 85% yield) as a white solid. ^1^H NMR (500 MHz, Chloroform-*d*) δ 7.31 (d, *J* = 7.5, 3.2 Hz, 1H), 3.13 (q, *J* = 7.6, 2.4 Hz, 2H), 1.44 (t, 3H), 0.28 (s, 9H). MS (ESI) m/z 296.1 [M+H]^+^.

#### 4,6-difluoro-2-isopropyl-5-((trimethylsilyl)ethynyl)benzo[d]thiazole (**31c**).

Compound **31c** was prepared by following a similar procedure to that of **31b**. Yield, 57%. 1H NMR (500 MHz, Chloroform-d) δ δ 7.32 (d, *J* = 7.5, 3.2 Hz, 1H), 3.48 – 3.39 (m, 1H), 1.47 (d, *J* = 6.9, 2.4 Hz, 6H), 0.29 (s, 9H). MS (ESI) m/z 310.1 [M+H]^+^.

#### 2-cyclobutyl-4,6-difluoro-5-((trimethylsilyl)ethynyl)benzo[d]thiazole (**31d**).

Compound **31d** was prepared by following a similar procedure to that of **31b**. Yield, 46%. ^1^H NMR (500 MHz, Chloroform-*d*) δ 7.33 (d, *J* = 7.8, 1.8 Hz, 1H), 4.00 – 3.90 (m, 1H), 2.57 – 2.49 (m, 2H), 2.49 – 2.40 (m, 2H), 2.18 – 2.08 (m, 1H), 2.04 – 1.97 (m, 1H), 0.29 (s, 9H). MS (ESI) m/z 322.1.1 [M+H]^+^.

#### 2-cyclopentyl-4,6-difluoro-5-((trimethylsilyl)ethynyl)benzo[d]thiazole (**31e**).

Compound **31e** was prepared by following a similar procedure to that of **31b**. Yield, 59%. ^1^H NMR (500 MHz, Chloroform-*d*) δ 7.32 (d, *J* = 7.8, 3.4, 1.6 Hz, 1H), 3.59 – 3.49 (m, 1H), 2.31 – 2.20 (m, 2H), 1.97 – 1.84 (m, 4H), 1.79 – 1.71 (m, 2H), 0.30 (s, 9H). MS (ESI) m/z 336.1.1 [M+H]^+^.

#### 1-(4-amino-5-((4,6-difluoro-2-methylbenzo[d]thiazol-5-yl)ethynyl)-8,9-dihydropyrazino[1′,2′:1,5]pyrrolo[2,3-d]pyrimidin-7(6H)-yl)prop-2-en-1-one (**8h**).

Compound **31a** (53 mg, 0.19 mmol) was dissolved in tetrabutylammonium fluoride (0.29 mL, 0.29 mmol, 1.0 M in THF) in a 25 mL round-bottom flask. The reaction mixture was stirred at room temperature for 2 h. The reaction mixture was diluted with water and extracted with ethyl acetate. The combined organic layers were washed with brine, dried over anhydrous sodium sulfate and concentrated to afford the crude product, which was used directly in the next step without further purification. Compound **18a** (60 mg, 0.16 mmol) was dissolved in *N,N*-dimethylformamide (2 mL). To this solution were added the crude product above, Pd(PPh_3_)_2_Cl_2_ (11 mg, 0.016 mmol), CuI (6 mg, 0.032 mmol), and Et_3_N (60 mg, 0.6 mmol). The reaction mixture was stirred at 70 °C for 6 h under a nitrogen atmosphere. The reaction was cooled to room temperature and diluted with water and extracted with ethyl acetate. The combined organic layers were concentrated under reduced pressure to give the crude product, which was further purified by silica gel column chromatography (MeOH/DCM = 8%) to afford compound **8h** (31.0 mg, 43% yield) as a white solid. ^1^H NMR (400 MHz, DMSO-*d*_6_) δ 8.17 (s, 1H), 7.98 (d, *J* = 8.7 Hz, 1H), 7.35 – 6.28 (m, 3H), 6.23 (d, *J* = 16.4 Hz, 1H), 5.81 (d, *J* = 10.4, 2.2 Hz, 1H), 5.11 – 4.94 (m, 2H), 4.20 – 4.01 (m, 4H), 2.82 (s, 3H). ^13^C NMR (151 MHz, DMSO) δ 169.89, 165.46, 158.31, 157.50, 153.69, 152.82, 149.41, 138.59, 137.95, 135.24, 128.96, 128.32, 105.30, 102.28, 99.81, 92.70, 88.56, 81.45, 42.65, 41.70, 40.54, 20.27. HRMS (ESI) for C_22_H_17_F_2_N_6_OS [M+H]^+^, calcd: 451.1147, found: 451.1153. HPLC purity: 95.13%.

#### 1-(4-amino-5-((2-ethyl-4,6-difluorobenzo[d]thiazol-5-yl)ethynyl)-8,9-dihydropyrazino[1′,2′:1,5]pyrrolo[2,3-d]pyrimidin-7(6H)-yl)prop-2-en-1-one (**8i**).

Compound **8i** was prepared by following a similar procedure to that of **8h**. ^1^H NMR (500 MHz, DMSO-*d*_6_) δ 8.14 (s, 1H), 7.99 (d, *J* = 6.7 Hz, 1H), 7.28 – 6.28 (m, 3H), 6.27 – 6.11 (m, 1H), 5.87 – 5.70 (m, 1H), 5.01 (d, *J* = 49.6 Hz, 2H), 4.17 – 3.96 (m, 4H), 3.14 – 3.08 (m, 2H), 1.42 – 1.27 (m, 3H). ^13^C NMR (151 MHz, DMSO) δ 175.94, 165.45, 158.31, 157.44, 153.91, 152.86, 149.45, 138.54, 137.42, 135.31, 128.97, 128.32, 105.44, 102.03, 99.88, 92.73, 88.48, 81.48, 42.66, 41.73, 40.53, 27.49, 13.70. HRMS (ESI) for C_23_H_19_F_2_N_6_OS [M+H]^+^, calcd: 465.1304, found: 465.1305. HPLC purity: 99.48%.

#### 1-(4-amino-5-((4,6-difluoro-2-isopropylbenzo[d]thiazol-5-yl)ethynyl)-8,9-dihydropyrazino[1′,2′:1,5]pyrrolo[2,3-d]pyrimidin-7(6H)-yl)prop-2-en-1-one (**8j**).

Compound **8j** was prepared by following a similar procedure to that of **8h**. ^1^H NMR (400 MHz, DMSO-*d*_6_) δ 8.19 (s, 1H), 8.05 (d, *J* = 8.7 Hz, 1H), 7.27 – 6.28 (m, 3H), 6.23 (d, *J* = 16.5 Hz, 1H), 5.81 (d, *J* = 10.8 Hz, 1H), 5.14 – 4.92 (m, 2H), 4.21 – 3.98 (m, 4H), 3.51 – 3.39 (m, 1H), 1.42 (d, *J* = 6.8 Hz, 6H). ^13^C NMR (151 MHz, DMSO) δ 179.54, 164.38, 157.23, 156.43, 152.95, 151.81, 148.41, 137.39, 136.02, 134.24, 127.89, 127.25, 104.44, 101.11, 98.88, 91.72, 87.44, 80.41, 41.59, 40.66, 39.46, 32.76, 21.75. HRMS (ESI) for C_24_H_21_F_2_N_6_OS [M+H]^+^, calcd: 479.1460, found: 479.1452. HPLC purity: 99.24%.

#### 1-(4-amino-5-((2-cyclopropyl-4,6-difluorobenzo[d]thiazol-5-yl)ethynyl)-8,9-dihydropyrazino[1′,2′:1,5]pyrrolo[2,3-d]pyrimidin-7(6H)-yl)prop-2-en-1-one (**8k**).

Compound **8k** was prepared from intermediate **24** by following a similar procedure to that of **8h**. ^1^H NMR (400 MHz, DMSO-*d*_6_) δ 8.16 (s, 1H), 7.99 (d, *J* = 8.6 Hz, 1H), 7.12 – 6.31 (m, 3H), 6.23 (d, *J* = 16.6 Hz, 1H), 5.81 (d, *J* = 11.0 Hz, 1H), 5.13 – 4.96 (m, 2H), 4.20 – 4.02 (m, 4H), 2.64 – 2.55 (m, 1H), 1.32 – 1.25 (m, 2H), 1.21 – 1.14 (m, 2H). ^13^C NMR (151 MHz, DMSO) δ 175.67, 164.37, 157.04, 156.40, 152.40, 151.85, 148.40, 137.50, 135.51, 134.22, 127.89, 127.26, 104.31, 100.90, 98.83, 91.66, 87.40, 80.44, 41.59, 40.65, 39.46, 14.23, 11.67. HRMS (ESI) for C_24_H_19_F_2_N_6_OS [M+H]^+^, calcd: 477.1304, found: 477.1309. HPLC purity: 97.59%.

#### 1-(4-amino-5-((2-cyclobutyl-4,6-difluorobenzo[d]thiazol-5-yl)ethynyl)-8,9-dihydropyrazino[1′,2′:1,5]pyrrolo[2,3-d]pyrimidin-7(6H)-yl)prop-2-en-1-one (**8l**).

Compound **8l** was prepared by following a similar procedure to that of **8h**. ^1^H NMR (500 MHz, DMSO-*d*_6_) δ 8.14 (s, 1H), 8.01 (d, *J* = 8.6 Hz, 1H), 7.50 – 6.25 (m, 3H), 6.20 (d, *J* = 16.1 Hz, 1H), 5.79 (d, *J* = 10.7 Hz, 1H), 5.09 – 4.96 (m, 2H), 4.17 – 4.04 (m, 4H), 4.03 – 3.99 (m, 1H), 2.47 – 2.42 (m, 2H), 2.40 – 2.32 (m, 2H), 2.11 – 2.01 (m, 1H), 1.97 – 1.89 (m, 1H). ^13^C NMR (151 MHz, DMSO) δ 176.89, 164.38, 157.24, 156.41, 152.96, 151.87, 148.42, 137.61, 136.32, 134.28, 127.89, 127.26, 104.50, 100.94, 98.92, 91.74, 87.38, 80.41, 41.60, 40.66, 39.46, 37.39, 28.33, 17.33. HRMS (ESI) for C_25_H_21_F_2_N_6_OS [M+H]^+^, calcd: 491.1460, found: 491.1466. HPLC purity: 98.96%.

#### 1-(4-amino-5-((2-cyclopentyl-4,6-difluorobenzo[d]thiazol-5-yl)ethynyl)-8,9-dihydropyrazino[1′,2′:1,5]pyrrolo[2,3-d]pyrimidin-7(6H)-yl)prop-2-en-1-one (**8m**).

Compound **8m** was prepared by following a similar procedure to that of **8h**. ^1^H NMR (500 MHz, DMSO-*d*_6_) δ 8.15 (s, 1H), 8.00 (d, *J* = 8.8 Hz, 1H), 7.35 – 6.27 (m, 3H), 6.20 (d, *J* = 16.3 Hz, 1H), 5.79 (d, *J* = 10.4 Hz, 1H), 5.08 – 4.94 (m, 2H), 4.17 – 4.00 (m, 4H), 3.64 – 3.54 (m, 1H), 2.22 – 2.12 (m, 2H), 1.90 – 1.81 (m, 2H), 1.81 – 1.72 (m, 2H), 1.72 – 1.64 (m, 2H). ^13^C NMR (151 MHz, DMSO) δ 178.01, 164.38, 157.23, 156.50, 152.87, 151.80, 148.43, 137.42, 136.16, 134.28, 127.89, 127.26, 104.40, 101.31, 98.85, 91.69, 87.49, 80.42, 43.28, 41.60, 40.66, 39.47, 32.72, 24.51. HRMS (ESI) for C_26_H_23_F_2_N_6_OS [M+H]^+^, calcd: 505.1617, found: 505.1622. HPLC purity: 99.44%.

#### (R)-1-(4-amino-5-((4,6-difluorobenzo[d]thiazol-5-yl)ethynyl)-8-methyl-8,9-dihydropyrazino[1′,2′:1,5]pyrrolo[2,3-d]pyrimidin-7(6H)-yl)prop-2-en-1-one (**8n**).

Compound **20b** (62 mg, 0.22 mmol) was dissolved in *N,N*-dimethylformamide (2 mL). To this solution were added compound **27** (80 mg, 0.27 mmol), Pd(PPh_3_)_2_Cl_2_ (15 mg, 0.022 mmol), CuI (8 mg, 0.044 mmol), and Et_3_N (111 mg, 1.1 mmol). The reaction mixture was stirred at 70 °C for 6 h under a nitrogen atmosphere. The reaction was cooled to room temperature and diluted with water and extracted with ethyl acetate. The combined organic layers were concentrated under reduced pressure to give the crude product, which was further purified by silica gel column chromatography (MeOH/DCM = 8%) to afford compound **8n** (55.4 mg, 56% yield) as a yellow solid. ^1^H NMR (400 MHz, DMSO-*d*_6_) δ 9.48 (s, 1H), 8.22 – 8.13 (m, 2H), 7.09 – 6.58 (m, 3H), 6.23 (d, *J* = 16.5 Hz, 1H), 5.82 (d, *J* = 10.2 Hz, 1H), 5.25 (d, *J* = 18.1 Hz, 1H), 4.97 – 4.82 (m, 1H), 4.74 – 4.55 (m, 1H), 4.40 – 4.34 (m, 1H), 4.19 – 4.07 (m, 1H), 1.15 – 1.06 (m, 3H). ^13^C NMR (151 MHz, DMSO) δ 165.45, 159.16, 158.80, 157.57, 154.88, 153.00, 149.83, 139.21, 136.86, 134.60, 128.79, 105.71, 102.16, 100.14, 92.80, 88.56, 81.61, 47.31, 45.81, 40.53, 18.00. HRMS (ESI) for C_22_H_17_F_2_N_6_OS [M+H]^+^, calcd: 451.1147, found: 451.1149. HPLC purity: 95.21%.

#### (R)-1-(4-amino-5-((4,6-difluoro-2-methylbenzo[d]thiazol-5-yl)ethynyl)-8-methyl-8,9-dihydropyrazino[1′,2′:1,5]pyrrolo[2,3-d]pyrimidin-7(6H)-yl)prop-2-en-1-one (**8o**).

Compound **8o** was prepared from intermediate **20b** by following a similar procedure to that of **8h**. ^1^H NMR (400 MHz, DMSO-*d*_6_) δ 8.18 (s, 1H), 8.03 (d, *J* = 8.81, 1.3 Hz, 1H), 7.48 – 6.30 (m, 3H), 6.23 (d, *J* = 16.6, 2.2 Hz, 1H), 5.81 (d, *J* = 10.4, 2.3 Hz, 1H), 5.24 (d, *J* = 18.2 Hz, 1H), 5.06 – 4.84 (m, 1H), 4.84 – 4.47 (m, 1H), 4.42 – 4.29 (m, 1H), 4.19 – 3.98 (m, 1H), 2.84 (s, 3H), 1.15 – 1.00 (m, 3H). ^13^C NMR (151 MHz, DMSO) δ 170.01, 165.45, 158.36, 157.57, 153.79, 152.96, 149.80, 138.65, 138.08, 134.45, 128.78, 105.38, 102.23, 99.84, 92.62, 88.62, 81.70, 47.35, 45.79, 40.53, 20.29, 17.97. HRMS (ESI) for C_23_H_19_F_2_N_6_OS [M+H]^+^, calcd: 465.1304, found: 465.1309. HPLC purity: 97.89%.

#### (R)-1-(4-amino-5-((2-(difluoromethyl)-4,6-difluorobenzo[d]thiazol-5-yl)ethynyl)-8-methyl-8,9-dihydropyrazino[1′,2′:1,5]pyrrolo[2,3-d]pyrimidin-7(6H)-yl)prop-2-en-1-one (**8p**).

Compound **8p** was prepared from intermediate **28a** by following a similar procedure to that of **8n**. ^1^H NMR (500 MHz, DMSO-*d*_6_) δ 8.20 (d, *J* = 8.7 Hz, 1H), 8.15 (s, 1H), 7.52 (t, *J* = 53.6, 1.7 Hz, 1H), 7.16 – 6.29 (m, 3H), 6.20 (d, *J* = 16.6 Hz, 1H), 5.79 (d, *J* = 10.6 Hz, 1H), 5.23 (d, *J* = 18.1 Hz, 1H), 4.99 – 4.81 (m, 1H), 4.77 – 4.48 (m, 1H), 4.38 – 4.32 (m, 1H), 4.15 – 4.03 (m, 1H), 1.12 – 1.01 (m, 3H). ^13^C NMR (151 MHz, DMSO) δ 165.45, 159.16, 158.80, 157.57, 154.88, 153.00, 149.83, 139.21, 136.86, 134.60, 128.79, 105.71, 102.16, 100.14, 92.80, 88.56, 81.61, 47.31, 45.81, 40.53, 18.00. HRMS (ESI) for C_23_H_17_F_4_N_6_OS [M+H]^+^, calcd: 501.1115, found: 501.1142. HPLC purity: 99.48%.

#### (R)-1-(4-amino-5-((4,6-difluoro-2-(trifluoromethyl)benzo[d]thiazol-5-yl)ethynyl)-8-methyl-8,9-dihydropyrazino[1′,2′:1,5]pyrrolo[2,3-d]pyrimidin-7(6H)-yl)prop-2-en-1-one (**8q**).

Compound **8q** was prepared from intermediate **28b** by following a similar procedure to that of **8n**. ^1^H NMR (600 MHz, DMSO-*d*_6_) δ 8.34 – 8.22 (m, 2H), 7.29 – 6.38 (m, 3H), 6.23 (d, *J* = 16.7 Hz, 1H), 5.81 (d, *J* = 10.6 Hz, 1H), 5.26 (d, *J* = 18.0 Hz, 1H), 5.03 – 4.83 (m, 1H), 4.84 – 4.43 (m, 1H), 4.39 – 4.35 (m, 1H), 4.21 – 4.02 (m, 1H), 1.13 – 1.07 (m, 3H). ^13^C NMR (151 MHz, DMSO) δ 170.01, 165.45, 158.36, 157.57, 153.79, 152.96, 149.80, 138.65, 138.08, 134.45, 128.78, 105.38, 102.23, 99.84, 92.62, 88.62, 81.70, 47.35, 45.79, 40.53, 20.29, 17.97. HRMS (ESI) for C_23_H_16_F_5_N_6_OS [M+H]^+^, calcd: 519.1021, found: 519.1022. HPLC purity: 95.15%.

#### (R)-1-(4-amino-5-((2-ethyl-4,6-difluorobenzo[d]thiazol-5-yl)ethynyl)-8-methyl-8,9-dihydropyrazino[1′,2′:1,5]pyrrolo[2,3-d]pyrimidin-7(6H)-yl)prop-2-en-1-one (**8r**).

Compound **8r** was prepared from intermediates **18b** and **31b** by following a similar procedure to that of **8h**. ^1^H NMR (400 MHz, DMSO-*d*_6_) δ 8.18 (s, 1H), 8.06 (d, *J* = 8.7 Hz, 1H), 7.19 – 6.38 (m, 3H), 6.23 (d, *J* = 16.7, 2.3 Hz, 1H), 5.82 (d, *J* = 9.4 Hz, 1H), 5.24 (d, *J* = 18.2 Hz, 1H), 4.99 – 4.82 (m, 1H), 4.79 – 4.52 (m, 1H), 4.41 – 4.34 (m, 1H), 4.18 – 4.02 (m, 1H), 3.17 (q, *J* = 7.5 Hz, 2H), 1.39 (t, *J* = 7.5 Hz, 3H), 1.14 – 1.02 (m, 3H). ^13^C NMR (151 MHz, DMSO) δ 174.92, 164.38, 157.27, 156.49, 152.85, 151.92, 148.74, 137.50, 136.44, 133.35, 127.71, 104.45, 101.09, 98.80, 91.60, 87.53, 80.65, 46.25, 44.74, 39.46, 26.43, 12.65. HRMS (ESI) for C_24_H_21_F_2_N_6_OS [M+H]^+^, calcd: 479.1460, found: 479.1463. HPLC purity: 98.76%.

#### (R)-1-(4-amino-5-((2-cyclopropyl-4,6-difluorobenzo[d]thiazol-5-yl)ethynyl)-8-methyl-8,9-dihydropyrazino[1′,2′:1,5]pyrrolo[2,3-d]pyrimidin-7(6H)-yl)prop-2-en-1-one (**8s**).

Compound **8s** was prepared from intermediates **18b** and **24** by following a similar procedure to that of **8h**. ^1^H NMR (400 MHz, DMSO-*d*_6_) δ 8.18 (s, 1H), 8.01 (d, *J* = 8.9 Hz, 1H), 7.12 – 6.31 (m, 3H), 6.23 (d, *J* = 16.6 Hz, 1H), 5.81 (d, *J* = 11.1 Hz, 1H), 5.23 (d, *J* = 18.4 Hz, 1H), 4.98 – 4.81 (m, 1H), 4.78 – 4.50 (m, 1H), 4.39 – 4.32 (m, 1H), 4.10 (s, 1H), 2.63 – 2.56 (m, 1H), 1.28 (d, *J* = 7.7 Hz, 2H), 1.21 – 1.15 (m, 2H), 1.13 – 1.03 (m, 3H). ^13^C NMR (151 MHz, DMSO) δ 175.70, 164.37, 157.05, 156.49, 152.43, 151.92, 148.74, 137.50, 135.57, 133.35, 127.70, 104.34, 101.11, 98.82, 91.57, 87.55, 80.70, 46.25, 44.70, 39.46, 16.88, 14.23, 11.66. HRMS (ESI) for C_25_H_21_F_2_N_6_OS [M+H]^+^, calcd: 491.1460, found: 491.1460. HPLC purity: 96.48%.

### Cell Culture.

SNU-16, BaF3, BaF3-EVT6-FGFR1, BaF3-EVT6-FGFR2 and BaF3-EVT6-FGFR3 were kindly provided by Jinan University. All cells mentioned above were cultured in RPMI 1640 medium. A204 and MDA-MB-453 cells were purchased from ATCC (USA). KATO III cells were obtained from Meisen Cell (China). BaF3-FGFR2^V564F^, BaF3 FGFR2^V564I^ and BaF3 FGFR2^N549K^ were cultured in RPMI 1640 medium. KATO III cells were cultured in RPMI 1640 medium. MDA-MB-453 cells were cultured in Leibovitz′s L-15 medium. A204 cells were cultured in complete McCoy′s 5A medium. All media were supplemented with 10% fetal bovine serum (FBS) and 1% streptomycin (P/S). All cell lines were cultured for less than 2 months after thaw and used within 20 passages. MDA-MB-453 cells are cultured at 37 °C in a carbon dioxide-free environment. All other cell lines mentioned above are cultured at 37 °C in a 5% CO_2_ environment.

### Western Blotting.

SNU-16 cells were treated with different concentrations of **KNT-0919** or DMSO (D8418-1L, CAS-No:67-87-5, Sigma-Aldrich) for 2 h, and the tested cells were washed by cold PBS buffer (Gibco) and lysed with 1X SDS buffer supplemented with complete protease inhibitor cocktail tablets (Sigma-Aldrich). The cell lysates were loaded and then electrophoresed to 10% SDS-PAGE gel, and the separated proteins were transferred to PVDF membranes. The proteins were transferred to a nitrocellulose (NC) membrane (Thermo Scientific) and the membranes were blocked in 5% milk buffer at room temperature for 1 h, followed by incubating with primary (1:1000) antibody at the recommended dilution at 4 °C for overnight. The membranes were washed by TBST for three times and incubated with anti-rabbit or anti-mouse HRP-conjugated secondary antibody for 1 h at room temperature, after which the membranes were imaged by Odyssey CLx Imager (LiCOR Biosciences). The protein signals were detected with Amersham Imager 800 system using a Western Lightning Plus-ECL (Perkin Elmer). Primary antibodies FGFR2 (23328S), phosphor-FGFR (3471S), phosphor-FRS2α (3861S), ERK1/2 (4695S), phosphor-ERK1/2 (4370S), Akt (pan) (11E7) (4685S), phosphor-AKT (9271S), GAPDH (5174), and secondary antibodies (1:2000) were purchased from Cell Signaling Technology (Boston, MA, USA). FSR2 (A-5) (sc-17841) was purchased from Santa Cruz Biotechnology (Dallas, TX, USA).

### FGFR Kinase Activity Inhibition Assay.

All the kinase activity was performed using an ADP-Glo kinase assay by Hefei PreceDo Medical Laboratory Co., Ltd. To a 384-well plate were added 2 μL of kinase and 1 μL of compound, and the mixture was incubated at room temperature for 5 min. Then, 2 μL of Substrate/ATP mixture was added, and the resulting mixture was incubated at room temperature for 60 min protected from light. Subsequently, 5 μL of ADP-Glo^™^ Reagent was added, followed by incubation at room temperature for 40 min. Next, 10 μL of Kinase Detection Reagent was added, and the mixture was incubated at room temperature for 30 min. The luminescence was recorded on a microplate reader with an integration time of 0.10 s.

### Kinetics of *K*_inact_ and *K*_i_.

The *K*_inact_ and *K*_i_ kinetics assay was conducted by Hefei PreceDo Medical Laboratory Co., Ltd (Project Code: PR-SHYJS-20260331). The irreversible inhibition kinetics of **KNT-0919** against FGFR1/2 were assessed using the ADP-Glo^™^ assay. Reactions were performed in kinase buffer (40 mM Tris-HCl, pH 7.5, 20 mM MgCl_2_, 0.01% Triton X-100, 1 mM DTT) at final concentrations of 0.02 ng/μL FGFR1 or 0.04 ng/μL FGFR2, 100 μM ATP, and 0.2 μg/μL polyE4Y1 substrate. In a 384-well plate, 1 μL enzyme and 3 μL serially diluted compound were incubated at room temperature for 0–1440 s. The kinase reaction was initiated with 4 μL substrate mixture and incubated for 60 min in the dark. Luminescence was measured on a PerkinElmer EnSight plate reader after quenching with ADP-Glo^™^ Reagent and detection with Kinase Detection Reagent. Residual activity was calculated relative to high (no inhibitor) and low (no enzyme) controls. Data were fitted to At=A0·e-kobs·t to obtain kobs values, which were then fitted to kobs= kinact·[I]/(Ki+[I]) to determine *K*_inact_ and *K*_i_.

### Kinase Selectivity Profiling of KNT-0919.

We use the KINOMEscan screening platform (ICE Bioscience Inc., China) to analyze **KNT-0919**, which was applied to a set of 416 kinases, covering protein kinase A, G, and C families (AGC), Ca2+/calmodul-independent protein kinase (CAMK), tyrosine kinase (TK), mitogen-activated protein kinase, glycogen synthase kinase, CDK-like kinase (CMGC), casein kinase I (CK1), and some atypical kinase families. In a visualization graph, the larger the red circle, the higher binding affinity of kinases. % Inhibition was calculated as follow: % Inhibition = 100% (compound-positive control)/(negative control-positive control) *100 %. The readout value of negative control (1 % DMSO) was set as a 0 % inhibition, and the readout value of background (10 μM Positive Control) was set as a 100 % inhibition.

### Protein Expression and Purification.

The kinase domains of FGFR2 (residues 465–765) and FGFR3 (residues 455–756, Δ574–585) were cloned into a pET-28a vector with an N-terminal 6 × His-tag followed by a PreScission protease cleavage site. The plasmids were co-transformed with a plasmid encoding YOPH phosphatase into *E. coli* BL21(DE3) cells. Protein expression was induced at an OD600 of ~0.6–0.8 by adding 0.5 mM IPTG, followed by incubation at 18°C for 18–20 h.

Cells were harvested by centrifugation, resuspended in lysis buffer (20 mM Tris-HCl pH 8.0, 500 mM NaCl, 20 mM imidazole, 1 mM TCEP), and lysed by a high-pressure homogenizer. The clarified lysate was loaded onto a Ni-NTA affinity column. After extensive washing with lysis buffer containing 40 mM imidazole, the His-tagged protein was eluted with lysis buffer containing 300 mM imidazole. The eluate was incubated with PreScission protease overnight at 4 °C to remove the His-tag. The cleavage mixture subsequently processed through anion exchange chromatography. Pure proteins were concentrated to approximately 10–20 mg/mL in the storage buffer (20 mM Tris-HCl, pH 8.0; 150 mM NaCl; 1 mM TCEP) and then flash frozen for storage at −80 °C for crystallization studies.

### Computational Modeling Studies.

The structure of FGFR2 was prepared using Protein Preparation Wizard and the compounds were prepared by LigPrep. The covalent docking was carried out using covalent docking module with default settings (Schrödinger, LLC, New York, NY, 2021).

### Protein Digestion and HPLC-Tandem MS (MS/MS) Analysis of Peptides.

Proteins were precipitated with acetone. The protein pellet was dried by using a Speedvac for 1–2 min. The pellet was subsequently dissolved in 8 M urea, 100 mM Tris-HCl, pH 8.5. TCEP (final concentration is 5 mM) (Thermo Scientific) and Iodoacetamide (final concentration is 10mM) (Sigma) for reduction and alkylation were added to the solution and incubated at room temperature for 30 minutes, respectively. The protein mixture was diluted four times and digested overnight with Trypsin at 1:50 (w/w) (Promega). The digested peptides were desalted using a home-packed C18 column and dried with a SpeedVac.

The peptide mixture was analyzed by a home-made 25 cm-long pulled-tip analytical column (75 μm ID packed with ReproSil-Pur C18-AQ 1.9 μm resin, Dr. Maisch GmbH), the column was then placed in-line with an Easy-nLC 1200 nano HPLC (Thermo Scientific) for mass spectrometry analysis. The analytical column temperature was set at 55 °C during the experiments. The mobile phase and elution gradient used for peptide separation were as follows: 0.1% formic acid in water as buffer A and 0.1% formic acid in 80% acetonitrile as buffer B, 0-1 min, 2%-10%B; 1-81 min, 10%-35% B; 81-96 min, 35-60% B; 96-111 min, 60%-100% B; 111-120 min, 100% B. The flow rate was set as 300 nl/min.

### Mass Spectrometry and Data Analysis.

Data-dependent MS/MS analysis was performed with a Q Exactive mass spectrometer (Thermo Scientific). Peptides eluted from the LC column were directly electrosprayed into the mass spectrometer with the application of a distal 2.2-kV spray voltage. A cycle of one full-scan MS spectrum (m/z 300-1800) was acquired followed by top 20 MS/MS events, sequentially generated on the first to the twentieth most intense ions selected from the full MS spectrum at a 28% normalized collision energy. Full scan resolution was set to 70,000 with automated gain control (AGC) target of 3e6. MS/MS scan resolution was set to 17,500 with isolation window of 1.8 m/z and AGC target of 5e5. The number of microscans was one for both MS and MS/MS scans and the maximum ion injection time was 50 and 100 ms, respectively. The dynamic exclusion settings used were as follows: charge exclusion, 1 and >7; exclude isotopes, on; and exclusion duration, 15 seconds. MS scan functions and LC solvent gradients were controlled by the Xcalibur data system (Thermo Scientific).

The acquired MS/MS data were analyzed against a Swiss-Prot *Homo Sapiens* database using PEAKS Studio 8.5 (Bioinformatics Solutions, Waterloo, Ontario, Canada). Precursor and product ion spectra were searched with an initial mass tolerance of 20 ppm and 0.02 Da, respectively. Semi-specific trypsin cleavage was selected, and up to three missed cleavages were allowed. Compound covalent binding on cysteine, phosphorylation on serine or threonine or tyrosine, carbamidomethylation on cysteine, and oxidation on methionine were set as variable modification. FDR was set as 1% and protein identification threshold was set as (−10 logP) ≥20. MSMS spectra of the modified peptides were manually confirmed and annotated.

### Crystallization and Structure Determination.

Purified kinase domains of FGFR2 and FGFR3 were incubated with compound **KNT-0919** at a molar ratio of 1:1.5 overnight at 4 °C. The protein-inhibitor complexes were subjected to initial crystallization screening kits at 20 °C *via* the sitting-drop vapor-diffusion method. Diffraction-quality crystals were subsequently optimized. Crystals of the FGFR2/**KNT-0919** complex were grown in a reservoir containing 0.2 M magnesium chloride, 0.1 M Bis-Tris pH 6.5, and 25% (w/v) PEG 3350, and appeared after approximately one month. Crystals of the FGFR3/**KNT-0919** complex were obtained within two weeks using a reservoir consisting of 0.2 M sodium chloride, 0.1 M HEPES pH 7.5, and 25% (w/v) PEG 3350.

For cryo-protection, crystals were briefly soaked in their respective mother liquors supplemented with 20% (v/v) glycerol before being flash-cooled in liquid nitrogen. X-ray diffraction data were collected at beamlines of the Shanghai Synchrotron Radiation Facility (SSRF). The structures were solved by molecular replacement using *PHENIX.Phaser*. For the FGFR2/**KNT-0919** complex, the previously determined structure (PDB: 9U7S) served as the search model. For the FGFR3/**KNT-0919** complex, the structure of a homologous kinase (PDB: 9VM9) was used as the search model. Iterative model building and refinement were performed in *Coot* and *PHENIX*, respectively. Data collection and refinement statistics are summarized in Table S2.

### Cell Proliferation Assay.

MDA-MB-453 were seeded in 384-well plates and incubated at 37 °C in a CO_2_-free environment. All other cell line were seeded in 384-well plates and incubated at 37 ℃ and 5% CO_2_. After overnight incubation, serial dilutions of tested compounds were added to the plate. The assay was tested with Cell Titer-Glo (Promega) after 3 days. The luminescence signal was detected by the Infinite M1000 Pro plate reader (Tecan), and the IC_50_ values were calculated by GraphPad Prism 10.

### Cell Apoptosis Assay.

A204 and SNU-16 cells were treated with indicated concentrations (0.1, 0.3, 1, 3, 10, 30, 100 nM) of our best candidate **KNT-0919** or DMSO (D8418-1L, CAS-No:67-87-5, Sigma-Aldrich) for 48 h at 37℃. After incubation, cells were collected and washed twice with pre-cold PBS. About 6 × 10^5^ cells were resuspended in 100 μl of 1× BD binding buffer solution, and then stained with 7-AAD and Annexin V-PE in the dark for 15 min. Finally, 400 μl of 1×BD binding buffer solution were added to stop dyeing. The cells were then measured on a BD FACSCelestaTM flow cytometer.

### Pharmacokinetics Study.

The pharmacokinetic investigation was taken by Hefei PreceDo Medical Laboratory Co., Ltd (Project Code: D-KET-2023010903). Specific pathogen free male and female ICR mice provided by Beijing Vital River Laboratory Animal Technology Co., Ltd. were dosed with tested compounds solution formulation (5% DMSO, 10% Solutol, 85% normal saline, 1 mg/kg for intravenous dose, 10 mg/kg for oral dose). Blood samples were collected at 0.083, 0.25, 0.5, 1, 2, 4, 8 and 24 h in intravenous administration, at 0.083, 0.25, 0.5, 1, 2, 4, 6, 8, and 24 h in oral administration. The blood samples were collected from sets of three mice at each time point in labeled microcentrifuge tubes containing heparin sodium as an anticoagulant. Plasma samples were separated by centrifugation (2-8 °C, 6000 rpm for 3 min) within 1 h and stored below −80 °C until bioanalysis. All samples were processed for analysis by precipitation using acetonitrile and analyzed with a partially validated LC/MS/MS method. Pharmacokinetic parameters were calculated using the noncompartmental analysis tool of Phoenix WinNonlin8.2.0 software.

### *In Vivo* Efficacy Study.

The animal experiments were performed following the protocol approved by University of Michigan (PRO00012191). Six- to eight-week-old CB17SCID female (Charles River Laboratory) mice were housed in a regular SPF room prior to cell injection. Briefly, 5 ×10^6^ cells of SNU-16 in 50% Matrigel (BD Biosciences) were injected subcutaneously into bilateral dorsal flanks of animals. After tumor size reached approximately 100–200 mm^3^, animals were randomized and subjected to drug treatment. Animals were administered with vehicle (5% DMSO, 10% Solutol, 85% Saline), 3, 10 or 30 mg/kg of compound **KNT-0919** by P.O. for 35 days. Tumor volume was calculated from digital caliper measurements made at the study outset, then twice weekly. Tumors were resected and weighed at the end of the time course.

### Histology Analysis.

Formalin-fixed paraffin embedded (FFPE) tumor sections were processed for hematoxylin and eosin (H&E) staining following standard procedures. Apoptosis was evaluated by Terminal dUTP Nick End Labeling (TUNEL) assay using In Situ Cell Death Detection Kit (#11684809910; Roche Applied Science) following the manufacturer’s instructions. Sections were deparaffinized, rehydrated, and treated with Proteinase K at 37 °C prior to incubation with the TUNEL reaction mixture. Signals were developed using DAB substrate. TUNEL-positive cells were quantified as the number of DAB-stained cells per 10 high-power fields (400×).

## Supplementary Material

si_001

Supporting Information

Kinetic parameters of **KNT-0919** for FGFR1 and FGFR2; Crystallization and structure determination of **KNT-0919**; Kinase selectivity profiling results of **KNT-0919**; Body weight change for *in vivo* study; The NMR spectra, HPLC and HRMS results of compounds **8a-s** (Word);

Molecular formula strings (CSV);

Predicted binding mode of **TAS-120** with FGFR2^V564F^ (PDB);

Predicted binding mode of **8a** with FGFR2 (PDB);

Predicted binding mode of **8a** with FGFR2 ^V564F^ (PDB);

Predicted binding mode of **8a** with FGFR3 (PDB);

Predicted binding mode of **8f** with FGFR2 (PDB).

## Figures and Tables

**Figure 1.**
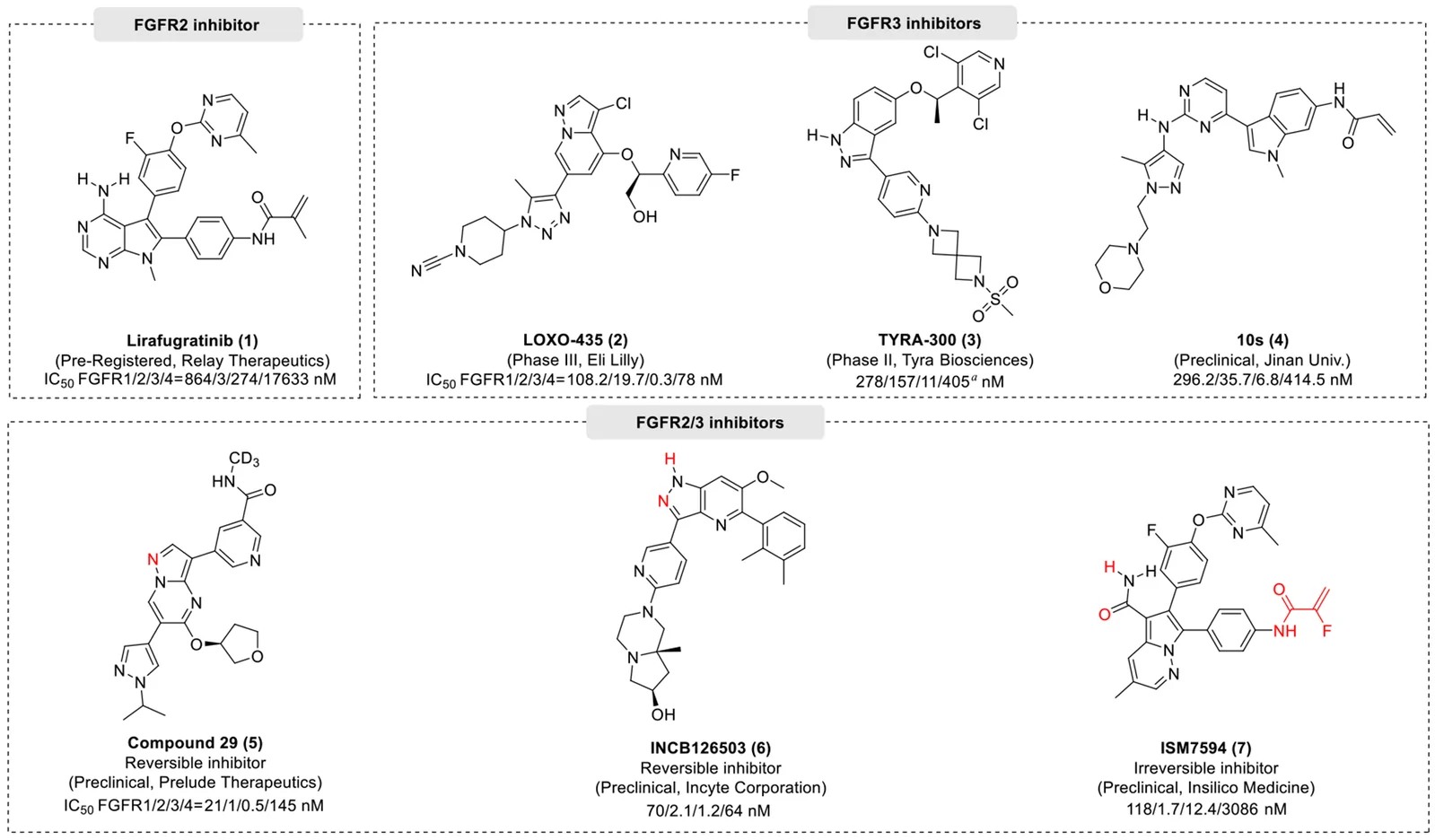
Chemical structures of representative selective FGFR2/3 inhibitors and their enzymatic inhibitory activities. ^*a*^Tested in Ba/F3 cell lines expressing FGFR1–4.

**Figure 2.**
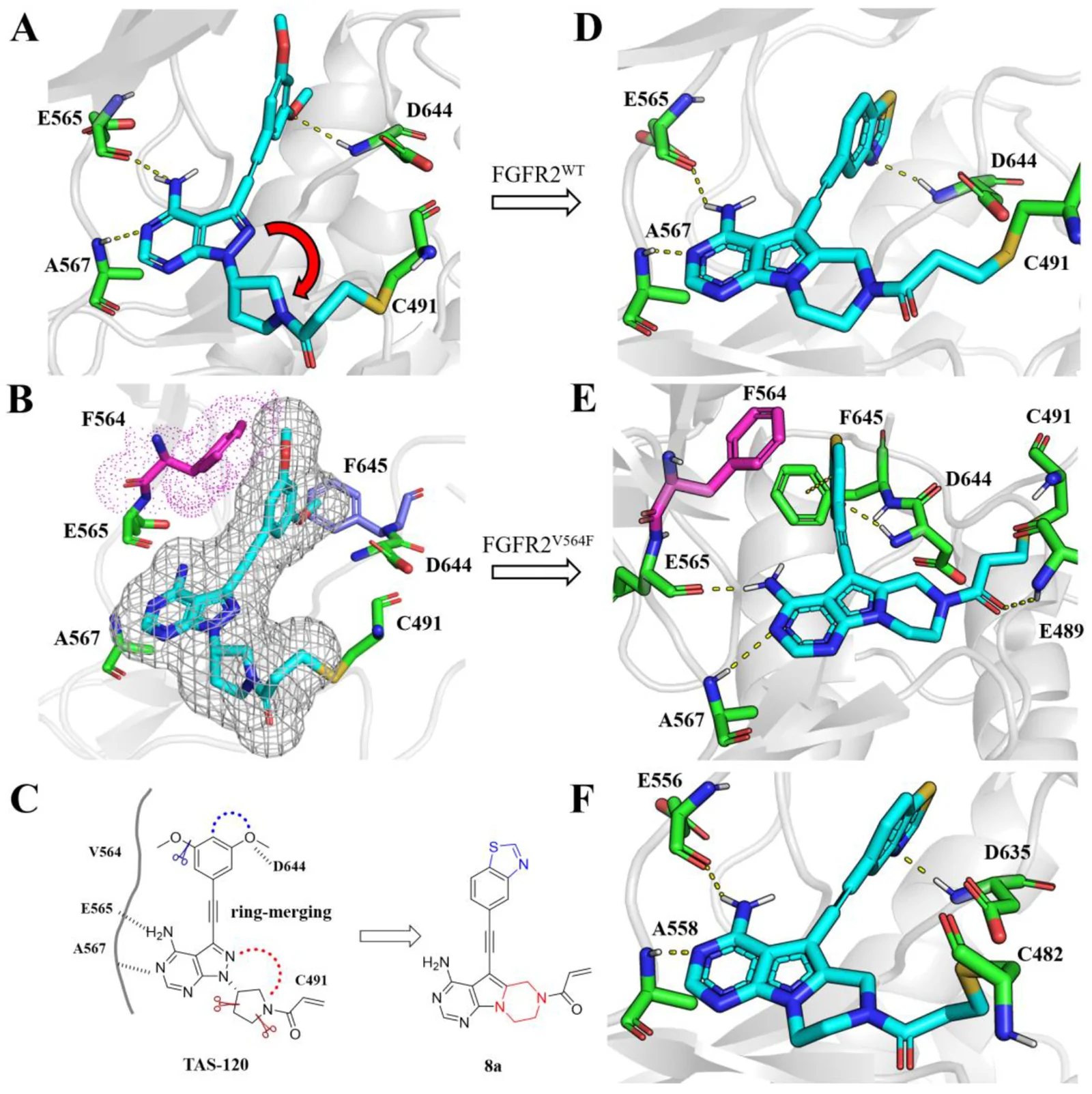
Design of new covalent FGFR2/3 inhibitors based on TAS-120. (A) Co-crystal structure of TAS-120 bound to FGFR2 (PDB: 8W2X); (B) The predicted binding mode of TAS-120 with FGFR2^V564F^; (C) Design of a novel covalent FGFR2/3 inhibitor **8a**; (D) The predicted binding mode of **8a** with FGFR2 (using PDB: 8W2X as the model); (E) The predicted binding mode of **8a** with FGFR2^V564F^ (using PDB: 9U3N as the model); (F) The predicted binding mode of **8a** with FGFR3 (using PDB: 8UDT as the model).

**Figure 3.**
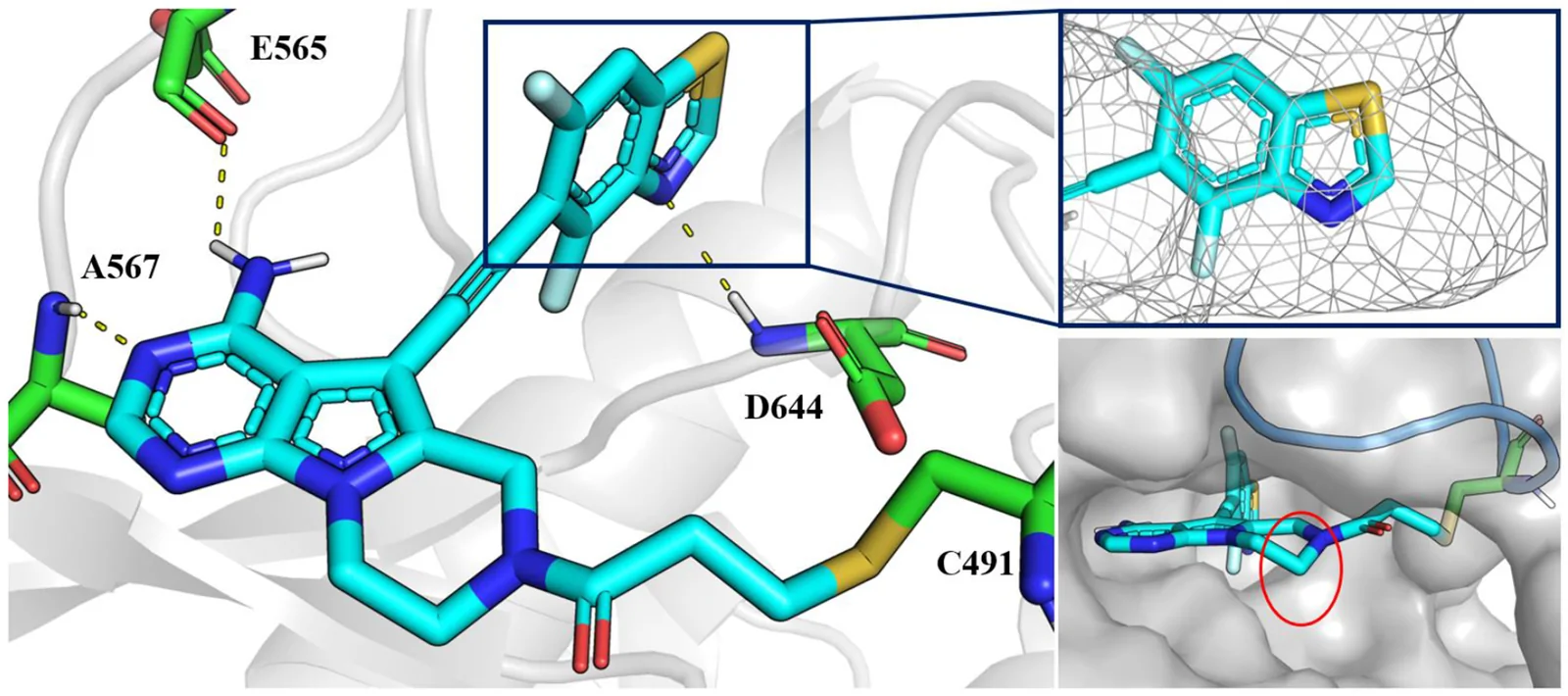
Predicted binding pose of **8f** in FGFR2 (using PDB: 8W2X as the model).

**Figure 4.**
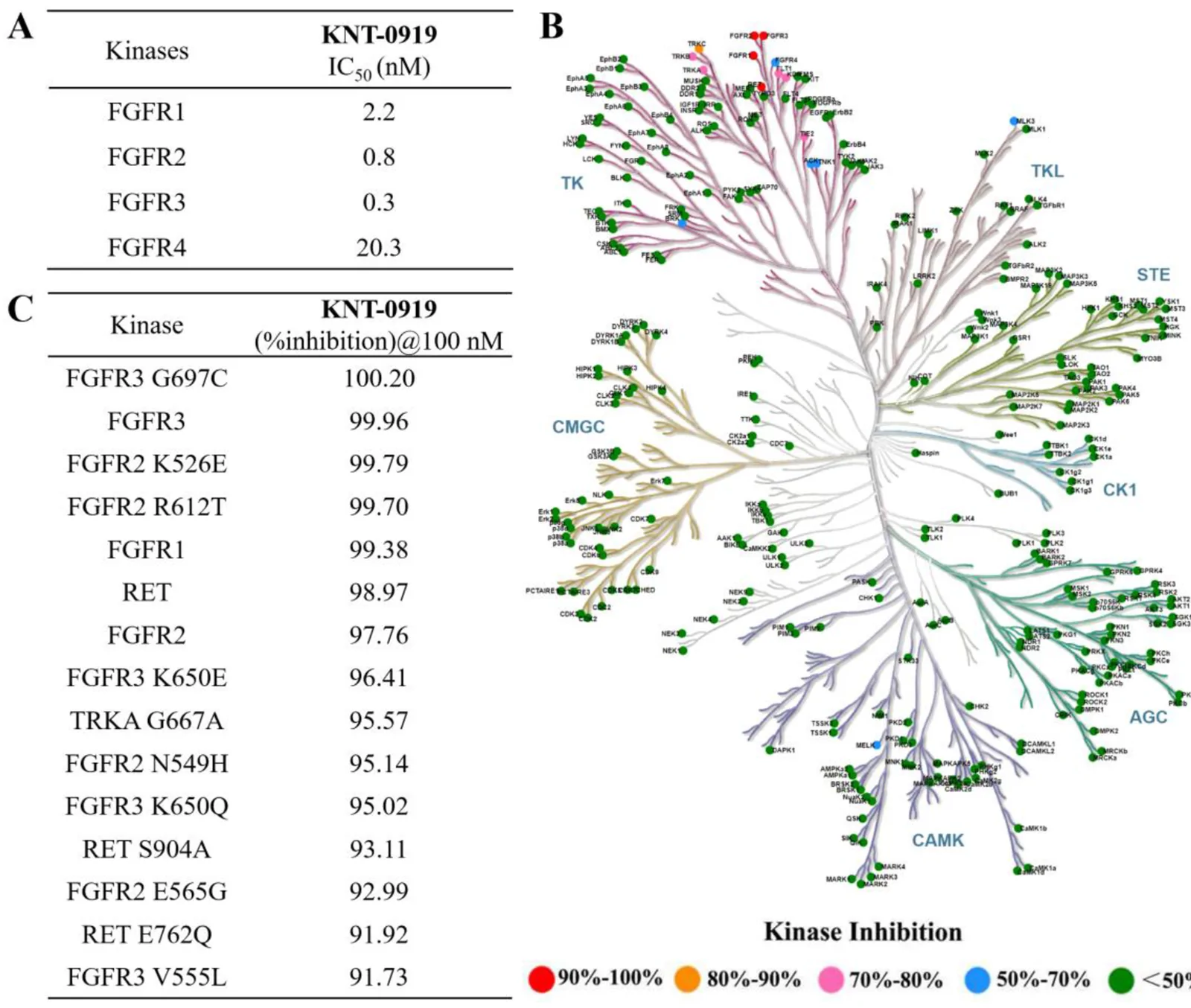
**KNT-0919** selectively inhibits FGFR2/3 kinase activity. (A) The IC_50_ values of **KNT-0919** against FGFR1-4; (B) Kinomescan profile of **KNT-0919** against 416 kinases at a concentration of 100 nM; (C) The kinases that showed the inhibition rate of >90 %.

**Figure 5.**
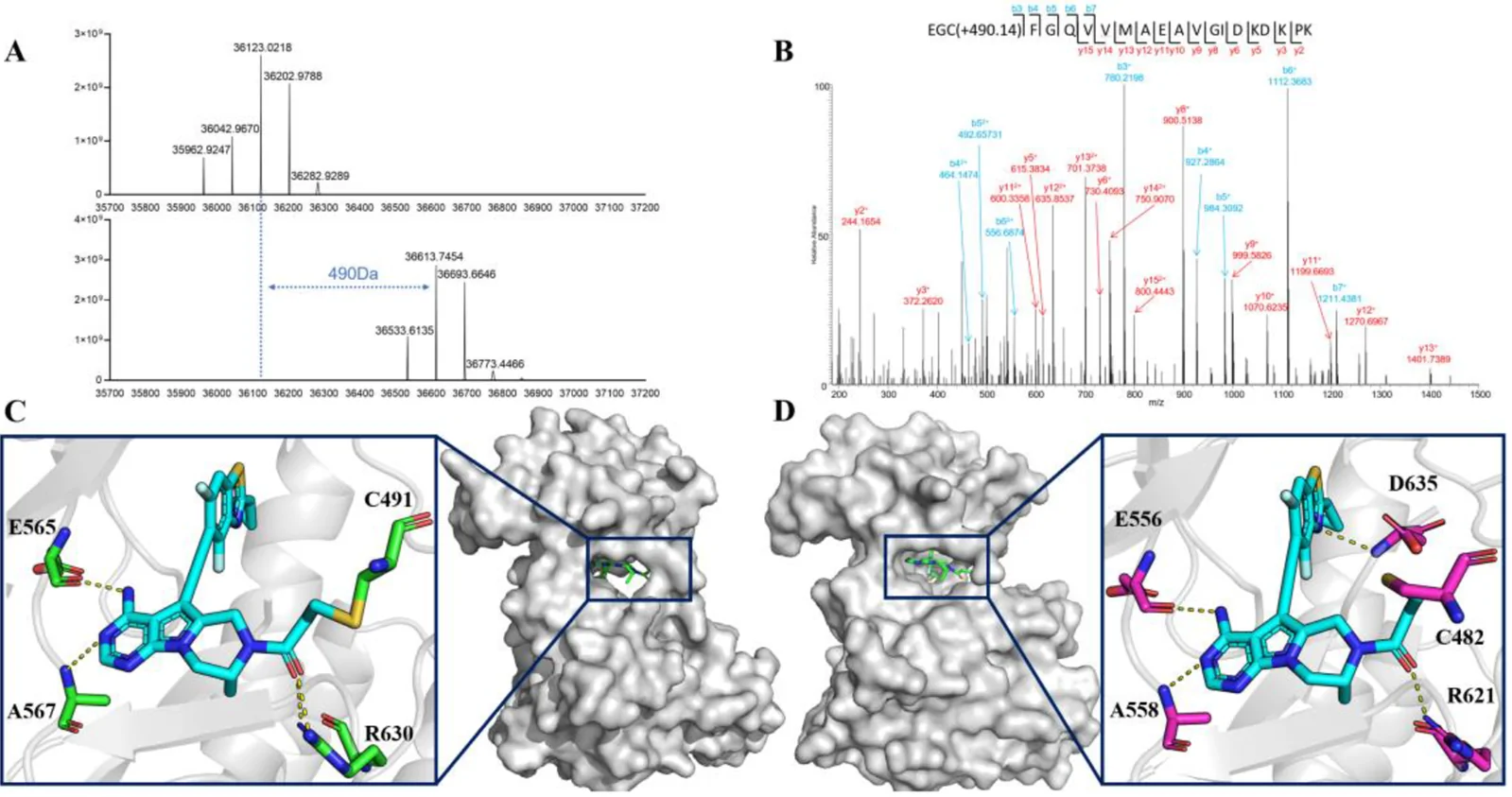
Covalent binding of **KNT-0919** to FGFR2/3. (A) Deconvoluted intact mass spectra of FGFR2 (top) and **KNT-0919**-labeled FGFR2 (bottom); (B) Higher energy collision-induced dissociation (HCD) MS/MS spectrum recorded on the [M+4H]^4+^ ion at m/z 678.3275 of the human FGFR2 peptide EGCFGQVVMAEAVGIDKDKPK harboring one modification site. Predicted b- and y-type ions (not including all) are listed above and below the peptide sequence, respectively. Matched ions are labeled in the spectrum and indicate that FGFR2 is modified on C491; (C) X-ray cocrystal structure of wild-type FGFR2 in complex with **KNT-0919** (PDB: 22ZR); (D) X-ray cocrystal structure of wild-type FGFR3 in complex with **KNT-0919** (PDB: 22ZS).

**Figure 6.**
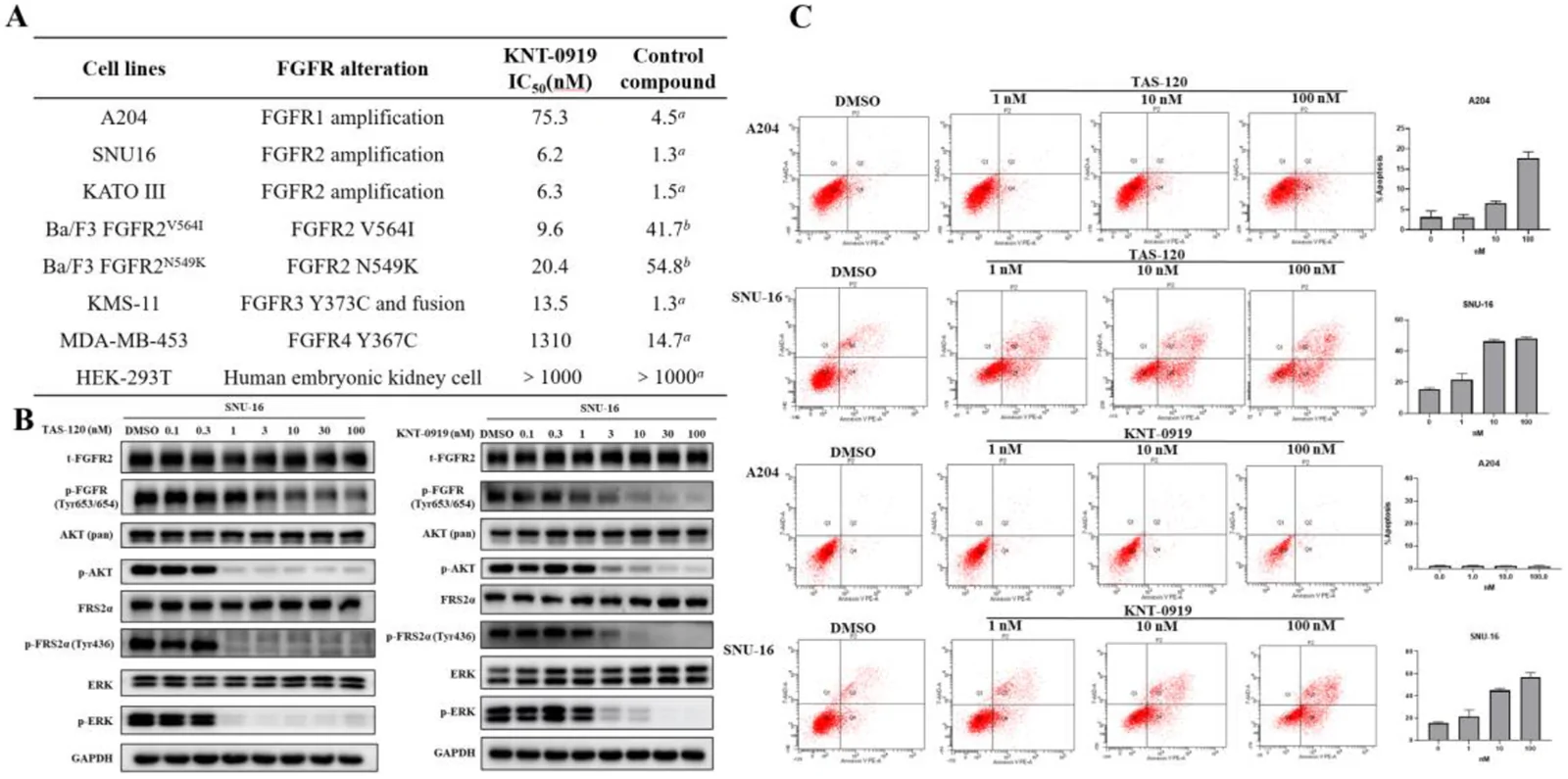
**KNT-0919** selectively suppresses FGFR2/3-dependent cancer cells proliferation and downstream signaling, and induces cell apoptosis. (A) The antiproliferation activities of **KNT-0919** against various cancer cell lines; (B) Inhibition of FGFR2-mediated signaling in SNU-16 cells; (C) **KNT-0919** selectively induced FGFR2-overexpressed cancer cell apoptosis. ^*a*^Tested with TAS-120. ^*b*^Tested with RLY-4008.

**Figure 7.**
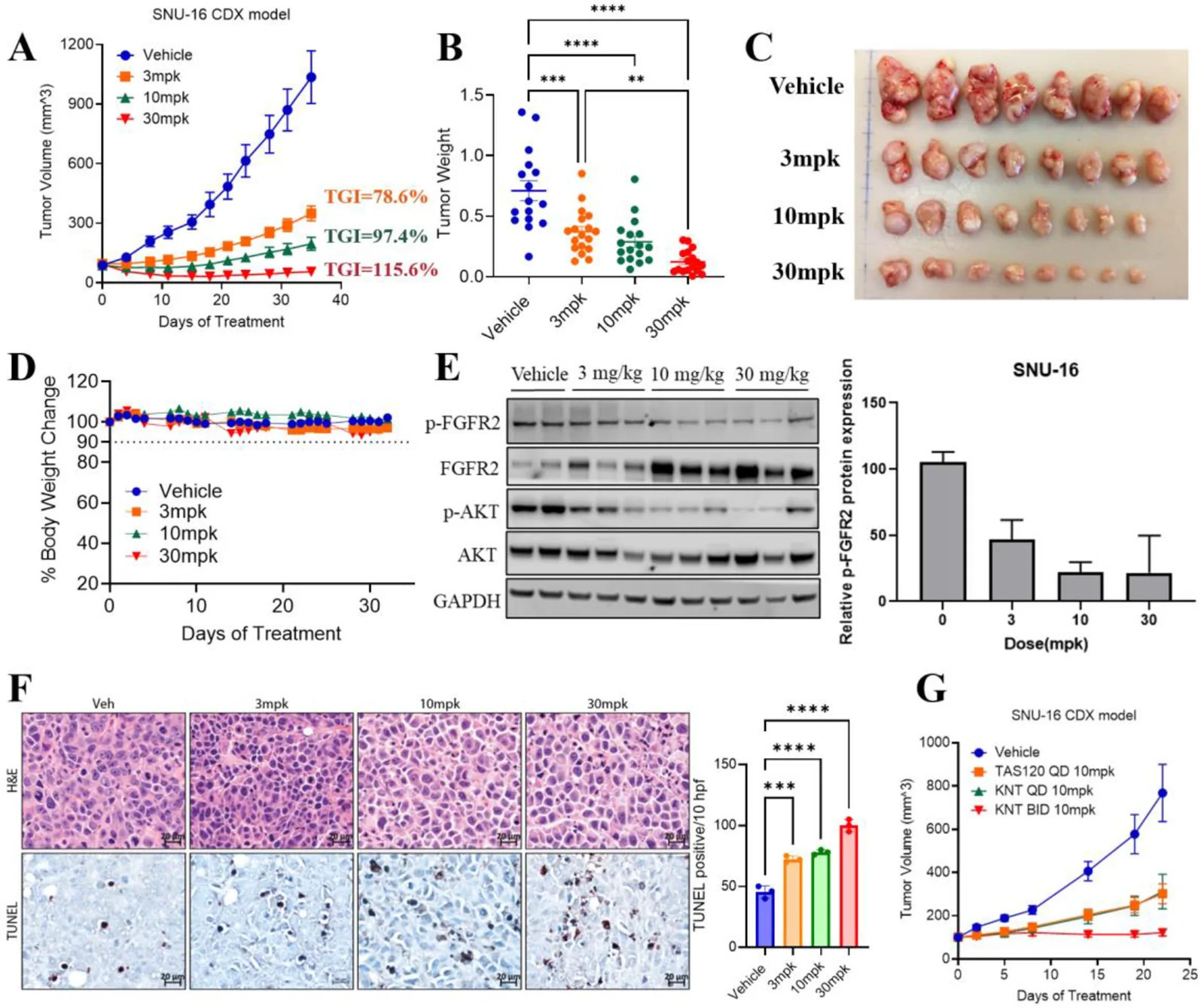
*In vivo* antitumor efficacy of **KNT-0919** in SNU-16 gastric cancer xenograft model. Tumor volume (A), tumor weights (B), tumor images (C) and mouse body weight (D) of SNU-16 tumor-bearing CB17SCID mice after p.o. administration of **KNT-0919** (3, 10 and 30 mg/kg, BID) for 35 days; (E) Dose-dependent suppression of FGFR2 signaling in tumors; (F) H&E and staining and TUNEL assay (left panel), and quantification of TUNEL assay (right panel) from **KNT-0919**-treated tumor tissues; (G) Tumor volume of SNU-16 tumor-bearing CB17SCID mice after p.o. administration of TAS-120 (10 mg/kg, QD) and **KNT-0919** (10 mg/kg, QD and BID) for 22 days.

**Scheme 1.**
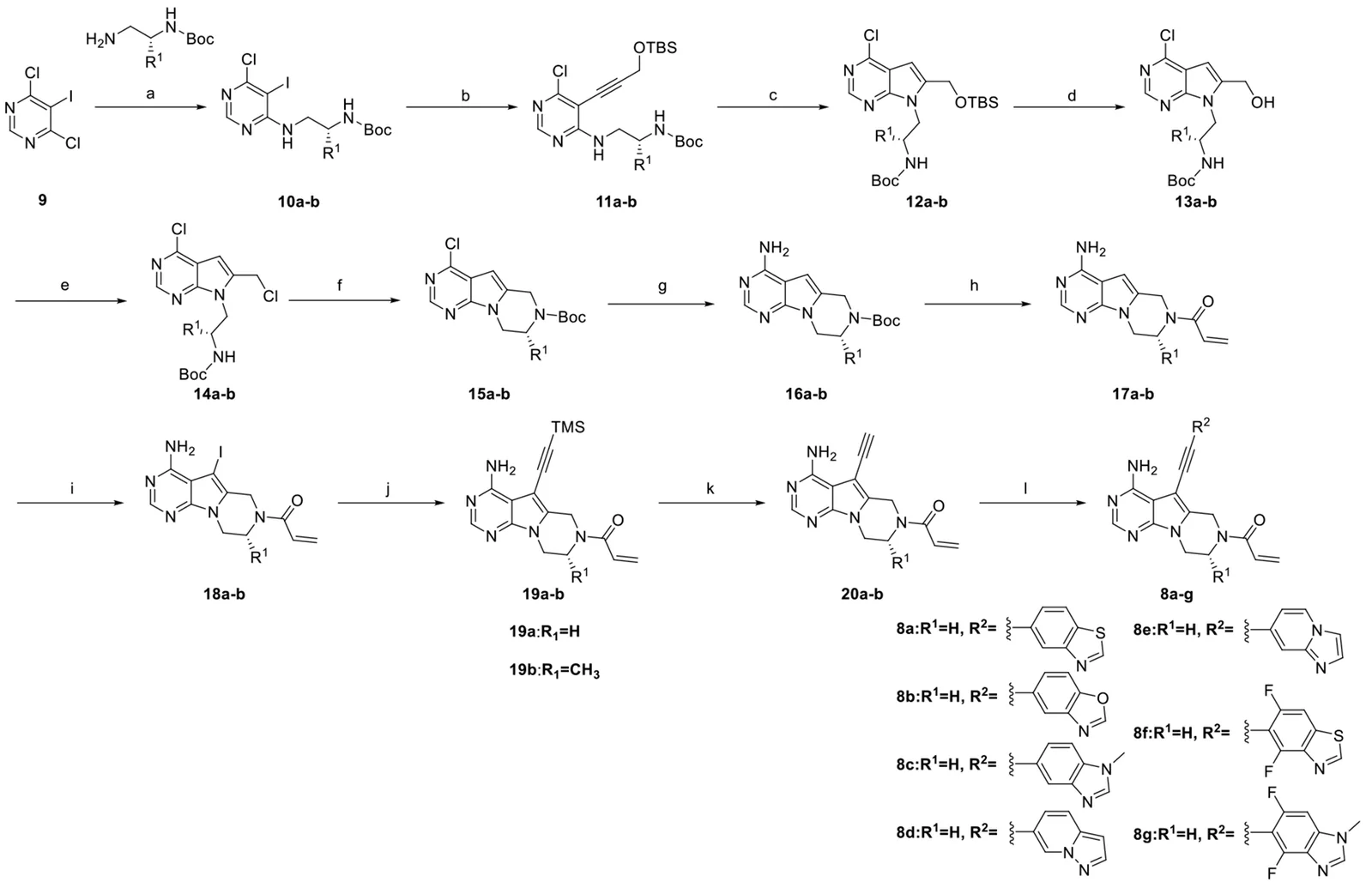
Synthesis of Compounds **8a-g**^a^ ^*a*^Reagents and conditions: (a) *N*, *N*-Diisopropylethylamine, CH_3_CN, room temperature (rt), overnight, 88-92%; (b) 3-(*tert*-Butyldimethylsilyloxy)-1-propyne, dichlorobis(triphenylphosphine)palladium(II), CuI, triethylamine (Et_3_N), tetrahydrofuran (THF), 70 °C, 6 h, 76-83%; (c) Cs_2_CO_3_, CH_3_CN, 90 °C, 6 h, 76-85%; (d) Tetrabutylammonium fluoride, THF, rt, 1 h; (e) Tosyl chloride, Et_3_N, CH_2_Cl_2_, rt, 3 h, 63-79% for two steps; (f) Cs_2_CO_3_, CH_3_CN, 70 °C, 10 h, 71-73%; (g) Aqueous ammonia, 1,4-dioxane, 120 °C, overnight, 45-49%; (h) 1) Trifluoroacetic acid, CH_2_Cl_2_, rt, 1 h; 2) Acrylic acid, propylphosphonic anhydride, Et_3_N, THF, rt, 36-57% for two steps; (i) *N*-Iodosuccinimide, THF, rt, 5 h, 65-79%; (j) Trimethylsilylacetylene, Pd(PPh_3_)_2_Cl_2_, CuI, Et_3_N, THF, 70 °C, 8 h, 68-71%; (k) tetrabutylammonium fluoride (TBAF) , THF, rt, 1 h; (l) Iodinated benzoxazole derivatives, Pd(PPh_3_)_2_Cl_2_, CuI, Et_3_N, *N,N*-dimethylformamide (DMF), 70 °C, 8 h, 34-64% for two steps.

**Scheme 2.**
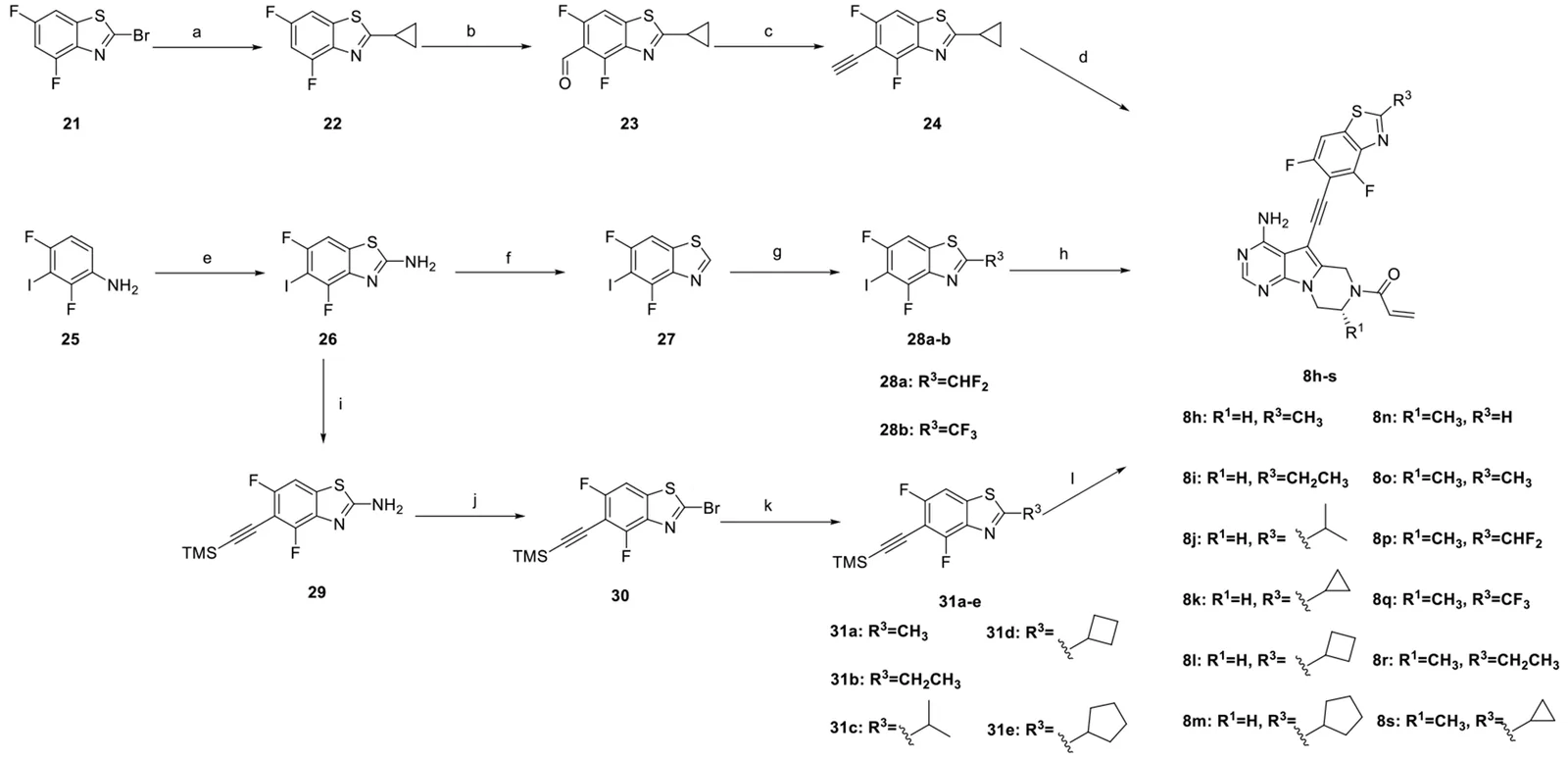
Synthesis of Compounds **8h–s**. ^a^ ^*a*^Reagents and conditions: (a) Cyclopropylboronic acid, Pd(dppf)Cl_2_, K_3_PO_4_, 1,4-dioxane, 100 °C, 8 h, 80%; (b) Lithium diisopropylamide, DMF, THF, −78 °C, 71%; (C) Dimethyl (1-diazo-2-oxopropyl)phosphonate, K_2_CO_3_, MeOH, rt, 73%; (d) **18a-b**, Pd(PPh_3_)_2_Cl_2_, CuI, Et_3_N, DMF, 70 °C, 8 h, 45-75%. (e) Ammonium thiocyanate, Br_2_, CH_3_COOH, rt, overnight, 62%; (f) *tert*-Butyl nitrite, 1,4-dioxane, 85 °C, 6 h, 74%; (g) 1) N_2_H_4_·H_2_O, EtOH, 80 °C, 1 h; 2) (R^3^CO)_2_O, *N*-methylpyrrolidone(NMP), 130 °C, 2 h, 14-36% for two steps; (h) **20b**, Pd(PPh_3_)_2_Cl_2_, CuI, Et_3_N, DMF, 70 °C, 8 h, 46-63%; (i) Trimethylsilylacetylene, Pd(PPh_3_)_2_Cl_2_, CuI, Et_3_N, DMF, 70 °C, 8 h, 65%; (j) CuBr_2_, *tert*-butyl nitrite, CH_3_CN, rt, 3 h, 87%; (k) for **31a**: Trimethylboroxine, Pd(dppf)Cl_2_, K_3_PO_4_, 1,4-dioxane, 100 °C, 8 h, 65%; for **31b-f**: Alkylzinc reagent, Pd(dppf)Cl_2_, 1,4-dioxane, 100 °C, 8 h, 46-85%; (l)**18a-b**, Pd(PPh_3_)_2_Cl_2_, CuI, Et_3_N, DMF, 70 °C, 8 h, 35-70%.

**Table 1.** The antiproliferation efficacies against parental BaF3, BaF3-FGFR1/2/3 and BaF3-FGFR2^V564F^ cell lines and FGFR2 enzymatic inhibitory activities of compounds **8a–m**.^a^

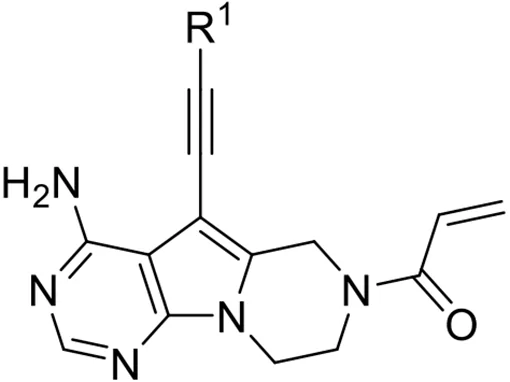							
Cpd.	R^1^	BaF3 (nM)	BaF3-FGFR1 (nM)	FGFR2 IC_50_ (nM)	BaF3-FGFR2 (nM)	BaF3-FGFR3 (nM)	BaF3-FGFR2^V564F^(nM)
**TAS-120**	/	> 1000	3.0	1.2	5.4	2.1	153
**8a**	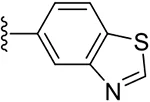	> 1000	337	39.0	7.8	21.2	89.8
**8b**	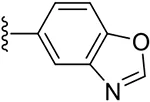	> 1000	> 1000	61.9	20.3	64.9	399
**8c**	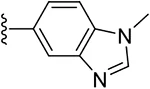	> 1000	726	46.6	12.9	59.9	286
**8d**	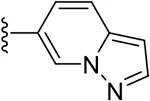	> 1000	> 1000	237	29.7	74.3	303
**8e**	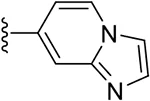	> 1000	> 1000	322	79.1	154	373
**8f**	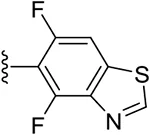	> 1000	215	16.2	6.4	6.8	29.1
**8g**	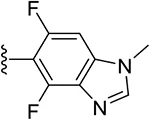	> 1000	964	46.4	13.5	27.8	46.6
**8h**	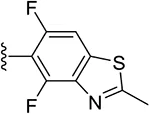	> 1000	132	19.0	6.6	3.6	33.8
**8i**	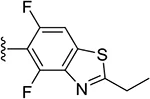	> 1000	200	17.9	17.7	12.4	75.4
**8j**	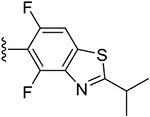	> 1000	327	39.2	60.1	49.9	253
**8k**	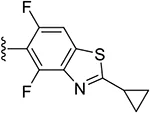	> 1000	464	17.1	27.8	28.0	68.0
**8l**	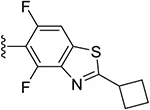	> 1000	297	33.4	51.6	60.4	130
**8m**	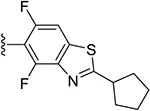	> 1000	296	46.1	49.2	92.2	92.5

a The data are mean values from at least two independent experiments.

**Table 2.** The antiproliferation efficacies against parental BaF3, BaF3-FGFR1/2/3 and BaF3-FGFR2^V564F^ cell lines and FGFR2 enzymatic inhibitory activities of compounds **8n–s**.^a^

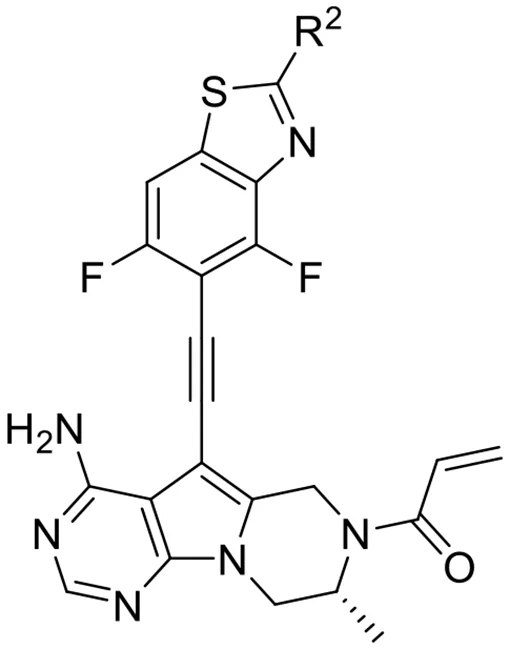							
Cpd.	R^2^	BaF3 (nM)	BaF3-FGFR1 (nM)	FGFR2 IC_50_ (nM)	BaF3-FGFR2 (nM)	BaF3-FGFR3 (nM)	BaF3-FGFR2^V564F^ (nM)
**8n**	H	> 1000	2.0	1.1	0.78	0.63	0.89
**8o**	CH_3_	> 1000	3.9	0.7	0.27	4.1	1.4
**8p**	CHF_2_	> 1000	4.9	2.3	0.92	0.38	9.4
**8q**	CF_3_	> 1000	6.3	4.5	1.2	0.40	20.8
**8r**	CH_2_CH_3_	> 1000	9.5	2.0	1.1	0.68	1.5
**8s (KNT-0919)**	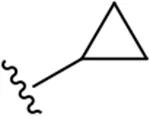	> 1000	17.1	0.8	1.1	0.20	5.6

a The data are mean values from at least two independent experiments.

**Table 3.** PK Parameters of Compound KNT-0919 in Rats.^a^

Route	T_1/2_ (h)	T_max_ (h)	C_max_ (ng/mL)	AUC_(0-t)_ (h*ng/mL)	AUC_(0-∞)_ (h*ng/mL)	CL (mL/min/kg)	F (%)
i.v. (1 mg/kg)	1.40	0.08	320	307	311	54	
p.o. (10 mg/kg)	1.52	0.67	500	1708	1787		55.6

a Data represent the geometric mean of three replicates.

## Data Availability

The reports of cocrystal structures of **KNT-0919** with FGFR2 (22ZR) and **KNT-0919** with FGFR3 (22ZS) will be released upon article publication.

## ABBREVIATIONS

AKT: protein kinase B
ATP: adenosine triphosphate
AUC: area under the curve
bid: twice a day
Boc: *tert*-butyloxycarbonyl
Br_2_: bromine
CH_2_Cl_2_: dichloromethane
CH_3_CN: acetonitrile
CH_3_COOH: acetic acid
CL: plasma clearance rate
C_max_: maximum drug plasma concentration
Cs_2_CO_3_: cesium carbonate
CuBr_2_: copper(II) bromide
CuI: copper(I) iodide
DMF: *N,N-*dimethylformamide
ERK: extracellular signal regulated kinase
Et_3_N: triethylamine
EtOH: ethanol
FGFR: fibroblast growth factor receptor
FRS2α: fibroblast growth factor receptor substrate 2 α
H&E: hematoxylin and eosin staining
HCD: higher-energy collision-induced dissociation
IC_50_: half maximal inhibitory concentration
IL-3: interleukin 3
K_3_PO_4_: potassium phosphate
LDA: lithium diisopropylamide
MAPK: RAS-mitogen-activated protein kinase
MeOH: methanol
MS: mass spectrometry
N_2_H_4_·H_2_O: hydrazine hydrate
NIS: *N-*iodosuccinimide
NMP: *N-*methylpyrrolidone
Pd(PPh_3_)_2_Cl_2_: bis(triphenylphosphine)palladium(II) chloride
Pd(dppf)Cl_2_: [1,1′-bis(diphenylphosphino)ferrocene]palladium(II) chloride
PI3K: phosphatidylinositol 3-kinase
PK: pharmacokinetic
PLCγ: phospholipase C γ
rt: room temperature
TBAF: tetrabutylammonium fluoride
TBS: *tert*-butyldimethylsilyl
TGI: tumor growth inhibition
THF: tetrahydrofuran
T_max_: time to maximum concentration
TMS: trimethylsilyl
t_1/2_: terminal half-life
TUNEL: terminal deoxynucleotidyl transferase dUTP nick end labeling
WB: western blot
