# Supplementary material for: Structure-based Design and Optimization of Novel, Potent and Selective Covalent FGFR2/3 Inhibitors with a Tricyclic Core
Source: J Med Chem. Author manuscript; Available in PMC 2026 Oct 7. (PMC13643868; doi:10.1021/acs.jmedchem.6c00585)
Supplement: Supplementary file 1 [file NIHMS2210484-supplement-Supporting_Information.docx]

**Supporting Information**

Structure-based Design and Optimization of Novel, Potent and Selective Covalent FGFR2/3 Inhibitors with a Tricyclic Core

Xuzhi Lu^1,2,#^, Zhaodi Tian^2,3,#^, Xueqiang Li^4,#^, Xiaojuan Chen^5,#^, Jean Ching-Yi Tien^6,7,#^, Yayuan Yang^1,2^, Yue Yin^8^, Qingrun Li^8^, Weixue Huang^2^, Fengtao Zhou^1^, Jinwei Zhang^2^, Xiaomei Ren^2^, Dan Liu^3^, Abigail J. Todd^6,7^, Shuqin Li^6,7^, Yongheng Chen^5,*^, Arul M. Chinnaiyan^6,7,9,10,11,*^, Shaohua Chang^4,*^, Ke Ding^1,2,*^, Zhen Wang^2,*^

^1^International Cooperative Laboratory of Traditional Chinese Medicine Modernization and Innovative Drug Discovery of Chinese Ministry of Education (MOE), Guangzhou City Key Laboratory of Precision Chemical Drug Development, College of Pharmacy, Jinan University, 855 Xingye Avenue East, Guangzhou 511400, China

^2^State Key Laboratory of Chemical Biology and Shanghai Hongkong Joint Laboratory in Chemical Synthesis, Shanghai Institute of Organic Chemistry, Chinese Academy of Sciences, #345 Lingling Rd., Shanghai 200032, China

^3^State Key Laboratory of Structure-Based Drugs Design & Discovery of Ministry of Education, Shenyang Pharmaceutical University, Shenyang 110016, China

^4^KinoTeck Therapeutics Co., Ltd, 35 Sicheng Road, Tianhe District, Guangzhou, 510663, China

^5^Department of Oncology, NHC Key Laboratory of Cancer Proteomics, State Local Joint Engineering Laboratory for Anticancer Drugs, Xiangya Hospital, Central South University, Changsha, Hunan 410008, China;

^6^Michigan Center for Translational Pathology, University of Michigan, Ann Arbor, Michigan 48109, USA

^7^Department of Pathology, University of Michigan, Ann Arbor, MI 48109, USA

^8^National Facility for Protein Science in Shanghai, Shanghai Advanced Research Institute, Chinese Academy of Science, Shanghai 201210, China

^9^Howard Hughes Medical Institute, University of Michigan, Ann Arbor, MI 48109, USA

^10^Rogel cancer Center, University of Michigan Ann Arbor, MI 48109, USA

^11^Department of Urology, University of Michigan, Ann Arbor, MI 48109, USA

^#^X.L., Z.T., X.L., X.C. and J.C.T. contributed equally to this work.

^*^Email: yonghenc@163.com (Y.C.); arul@med.umich.edu (A.M.C.); csh@kino-biotech.com (S.C.); dingk@sioc.ac.cn (K.D.); wangz@sioc.ac.cn (Z.W.).

**Table of Contents**

1. Kinetic Parameters of **KNT‑0919** for FGFR1 and FGFR2.............................................S3

2. Crystallization and structure determination of compound **KNT-0919**...............S3-S4

3. Kinase selectivity profiling of compound **KNT-0919**......................................S4-S14

4. The NMR spectra, HPLC and HRMS data of compounds **8a**-**s**.....................S15-S52

Table S1. Kinetic Parameters of **KNT‑0919** for FGFR1 and FGFR2

| **Target** | **K_i_ (nM)** | **K_inact_ (s^-1^)** | **K_inact_/K_i_ (nM^-1^·s^-1^)** |
| --- | --- | --- | --- |
| FGFR1 | 5.3 | 0.00599 | 0.00113 |
| FGFR2 | 0.4 | 0.00405 | 0.01053 |

Table S2. Data collection and refinement statistics

|  | FGFR2/**KNT-0919** | FGFR3/ **KNT-0919** |
| --- | --- | --- |
| **PDB code** | **22ZR** | **22ZS** |
| **Data collection** |  |  |
| Space group | P 1 21 1 | P 1 21 1 |
| Cell dimensions |  |  |
| *a*, *b*, *c* (Å) | 69.39, 58.11, 74.13 | 51.15, 84.72, 134.06 |
| α, β, γ (°) | 90.00, 108.25, 90.00 | 90.00, 90.29, 90.00 |
| Resolution (Å) | 30.66-3.15 (3.26-3.15) | 31.34-2.80 (2.90-2.80) |
| *R*_merge_ | 0.36 (1.28) | 0.71 (3.40) |
| CC(1/2) | 0.93 (0.11) | 0.97 (0.29) |
| Mean I/sigma(I) | 3.63 (1.33) | 5.45 (1.38) |
| Completeness (%) | 83.67 (35.91) | 86.00 (21.86) |
| Redundancy | 3.90 (2.60) | 5.80 (5.30) |
|  |  |  |
| **Refinement** |  |  |
| Unique reflections | 9359 (349) | 28333 (614) |
| *R*_work_/*R*_free_ | 0.27/0.31 | 0.24/0.28 |
| No. atoms |  |  |
| Macromolecules | 4242 | 8734 |
| Ligands | 70 | 140 |
| *B*-factors |  |  |
| Macromolecules | 56.80 | 39.42 |
| Ligands | 52.16 | 39.61 |
| R.m.s. deviations |  |  |
| Bond lengths (Å) | 0.165 | 0.16 |
| Bond angles (°) | 3.69 | 3.58 |

Table S3: Kinomescan data of **KNT-0919**

| Kinase | KNT-0919  (100 nM) | KNT-0919  (100 nM) | STDEV | KNT-0919 (%inhibition)  @100 nM |
| --- | --- | --- | --- | --- |
| FGFR3 G697C | 100.41 | 99.99 | 0.30 | 100.20 |
| FGFR3 | 99.91 | 100.01 | 0.07 | 99.96 |
| FGFR2 K526E | 99.67 | 99.90 | 0.16 | 99.79 |
| FGFR2 R612T | 99.64 | 99.76 | 0.08 | 99.70 |
| FGFR1 | 99.24 | 99.51 | 0.19 | 99.38 |
| RET | 98.59 | 99.34 | 0.53 | 98.97 |
| FGFR2 | 97.99 | 97.53 | 0.33 | 97.76 |
| FGFR3 K650E | 97.01 | 95.80 | 0.86 | 96.41 |
| TRKA G667A | 96.25 | 94.90 | 0.95 | 95.57 |
| FGFR2 N549H | 94.96 | 95.31 | 0.25 | 95.14 |
| FGFR3 K650Q | 95.68 | 94.35 | 0.94 | 95.02 |
| RET S904A | 92.75 | 93.46 | 0.50 | 93.11 |
| FGFR2 E565G | 93.86 | 92.13 | 1.23 | 92.99 |
| RET E762Q | 92.51 | 91.34 | 0.83 | 91.92 |
| FGFR3 V555L | 91.96 | 91.50 | 0.33 | 91.73 |
| TRKC | 91.01 | 88.76 | 1.59 | 89.89 |
| FGFR2 K659N | 89.02 | 88.47 | 0.38 | 88.75 |
| FGFR2 C491S | 90.07 | 87.22 | 2.01 | 88.65 |
| RET L730I | 84.75 | 85.10 | 0.25 | 84.92 |
| NCOA4-RET | 83.01 | 84.17 | 0.83 | 83.59 |
| TPR-TRKA | 83.36 | 83.56 | 0.14 | 83.46 |
| TRKA G667C | 81.71 | 82.79 | 0.76 | 82.25 |
| FGFR2 C491F | 83.38 | 79.72 | 2.59 | 81.55 |
| TRKC G696A | 80.11 | 80.74 | 0.45 | 80.43 |
| TRKA G667S | 80.67 | 76.80 | 2.74 | 78.74 |
| FGFR2 K641R | 83.03 | 73.94 | 6.43 | 78.48 |
| TRKB | 79.13 | 77.73 | 0.99 | 78.43 |
| RET Y806H | 77.70 | 78.36 | 0.46 | 78.03 |
| TFG-TRKA | 80.38 | 74.84 | 3.91 | 77.61 |
| FLT1 | 77.60 | 76.30 | 0.93 | 76.95 |
| RET M918T | 79.99 | 73.36 | 4.68 | 76.67 |
| TRKC L686M | 77.88 | 74.98 | 2.05 | 76.43 |
| TRKA | 79.54 | 73.15 | 4.51 | 76.35 |
| KDR | 76.19 | 76.50 | 0.22 | 76.34 |
| CCDC6-RET | 77.38 | 73.58 | 2.69 | 75.48 |
| FGFR2 V564F | 79.85 | 67.18 | 8.96 | 73.51 |
| BTK T474I | 72.51 | 71.79 | 0.51 | 72.15 |
| TIE2 | 74.63 | 69.38 | 3.71 | 72.01 |
| FGFR4 N535K | 72.30 | 66.39 | 4.18 | 69.35 |
| RET L730M | 73.84 | 64.01 | 6.96 | 68.93 |
| FGFR3 K650M | 70.04 | 62.77 | 5.14 | 66.41 |
| MLK3 | 61.63 | 66.31 | 3.31 | 63.97 |
| RET R921P | 63.40 | 63.80 | 0.29 | 63.60 |
| TNK1 | 63.13 | 63.92 | 0.56 | 63.52 |
| RET G691S | 67.70 | 56.94 | 7.61 | 62.32 |
| ACK | 55.25 | 61.23 | 4.23 | 58.24 |
| MELK | 55.35 | 59.18 | 2.70 | 57.26 |
| RET V804L | 50.90 | 62.17 | 7.97 | 56.54 |
| TRKC G623E | 56.72 | 55.98 | 0.52 | 56.35 |
| BRK | 55.88 | 55.51 | 0.26 | 55.70 |
| FGFR4 | 57.14 | 53.22 | 2.77 | 55.18 |
| TRKA G595R A608D | 57.12 | 49.32 | 5.52 | 53.22 |
| TRKA G595R | 54.96 | 49.21 | 4.06 | 52.09 |
| FGFR4 V550L | 48.00 | 51.82 | 2.70 | 49.91 |
| FGFR2 V564I | 50.94 | 47.59 | 2.37 | 49.27 |
| FLT3 ITD | 49.56 | 48.30 | 0.89 | 48.93 |
| RET S904F | 49.29 | 47.12 | 1.54 | 48.20 |
| TRKA A608D | 35.35 | 59.64 | 17.18 | 47.49 |
| MLK2 | 45.16 | 44.61 | 0.39 | 44.88 |
| FGFR3 V555M | 45.50 | 41.93 | 2.53 | 43.72 |
| FAK | 44.31 | 43.00 | 0.93 | 43.66 |
| PDGFRβ | 45.84 | 39.53 | 4.46 | 42.69 |
| FYN [isoform a] | 48.94 | 35.26 | 9.67 | 42.10 |
| PDGFRα V561D | 45.25 | 36.68 | 6.06 | 40.96 |
| BLK | 39.99 | 41.66 | 1.18 | 40.83 |
| CRAF | 40.09 | 41.41 | 0.93 | 40.75 |
| FLT4 | 48.42 | 33.00 | 10.90 | 40.71 |
| LRRK2 | 44.95 | 36.24 | 6.16 | 40.59 |
| RET G810R | 46.18 | 34.75 | 8.08 | 40.47 |
| PRKAR1A-RET | 48.60 | 31.64 | 11.99 | 40.12 |
| DDR1 | 30.83 | 45.02 | 10.03 | 37.93 |
| KIT T670I | 40.87 | 33.98 | 4.87 | 37.42 |
| GLK | 37.34 | 36.72 | 0.44 | 37.03 |
| RET V804M | 34.92 | 36.70 | 1.26 | 35.81 |
| IKKε | 37.56 | 33.45 | 2.91 | 35.51 |
| CK1ε | 35.73 | 34.81 | 0.65 | 35.27 |
| AurA | 33.78 | 35.92 | 1.52 | 34.85 |
| MAP3K3 | 30.11 | 39.15 | 6.40 | 34.63 |
| CSF1R | 45.63 | 23.19 | 15.87 | 34.41 |
| FLT3 | 36.18 | 30.84 | 3.78 | 33.51 |
| NuaK1 | 35.10 | 30.36 | 3.35 | 32.73 |
| INSR | 30.15 | 32.33 | 1.54 | 31.24 |
| HIPK2 | 33.98 | 25.76 | 5.82 | 29.87 |
| KHS1 | 29.08 | 29.94 | 0.61 | 29.51 |
| MET Y1230C | 31.50 | 26.78 | 3.34 | 29.14 |
| FGR | 30.21 | 27.45 | 1.95 | 28.83 |
| FGFR4 V550M | 29.91 | 27.00 | 2.05 | 28.45 |
| TGFβR1 T204D | 17.09 | 39.03 | 15.51 | 28.06 |
| ZAK | 19.88 | 33.40 | 9.57 | 26.64 |
| HGK | 29.84 | 21.19 | 6.12 | 25.52 |
| KIT | 33.56 | 16.67 | 11.94 | 25.11 |
| MAP4K2 | 23.15 | 26.80 | 2.58 | 24.98 |
| PEK | 16.06 | 30.83 | 10.45 | 23.44 |
| TGFβR1 | 37.65 | 8.77 | 20.42 | 23.21 |
| RET L790F | 24.72 | 21.55 | 2.25 | 23.14 |
| TRKA G595R G667A | 9.79 | 35.77 | 18.37 | 22.78 |
| MUSK | 24.49 | 19.64 | 3.43 | 22.07 |
| CLK4 | 22.15 | 21.64 | 0.36 | 21.90 |
| TNIK | 21.48 | 22.27 | 0.56 | 21.88 |
| CaMK1α | 24.15 | 19.22 | 3.49 | 21.68 |
| JNK2 | 18.46 | 24.83 | 4.50 | 21.64 |
| TTK | 29.90 | 12.15 | 12.55 | 21.02 |
| Nuak2 | 24.10 | 17.82 | 4.45 | 20.96 |
| AMPKα2/β2/γ3 | 23.82 | 18.02 | 4.10 | 20.92 |
| PLK4 | 28.69 | 12.92 | 11.16 | 20.80 |
| MAPKAPK5 | 22.14 | 19.35 | 1.97 | 20.75 |
| MSK1 | 23.86 | 16.83 | 4.97 | 20.34 |
| p38δ | 16.91 | 23.27 | 4.50 | 20.09 |
| MAP2K7 | 18.68 | 20.96 | 1.61 | 19.82 |
| NEK2 | 20.00 | 19.30 | 0.50 | 19.65 |
| CK2α2/β | 25.13 | 13.50 | 8.22 | 19.31 |
| MAP3K2 | 17.67 | 20.83 | 2.23 | 19.25 |
| MET D1228H | 18.05 | 19.30 | 0.88 | 18.68 |
| EPHA7 | 24.89 | 11.39 | 9.54 | 18.14 |
| HCK | 13.09 | 22.19 | 6.44 | 17.64 |
| ALK4 | 12.98 | 21.77 | 6.22 | 17.37 |
| ErK1 | 17.91 | 16.70 | 0.86 | 17.31 |
| IRR | 10.23 | 23.91 | 9.67 | 17.07 |
| CK2α1/β | 19.62 | 14.35 | 3.73 | 16.98 |
| JNK3 | 19.57 | 13.89 | 4.02 | 16.73 |
| LYNb | 16.01 | 17.27 | 0.89 | 16.64 |
| DYRK3 | 28.62 | 4.59 | 16.99 | 16.60 |
| MAP2K1 | 17.18 | 15.14 | 1.45 | 16.16 |
| CDK6/CyCD3 | 17.11 | 14.97 | 1.51 | 16.04 |
| CK1γ1 | 14.56 | 17.51 | 2.08 | 16.04 |
| AMPKα2/β2/γ2 | 27.58 | 4.39 | 16.40 | 15.98 |
| DYRK1B | 22.20 | 8.81 | 9.47 | 15.51 |
| RSK2 | 17.27 | 13.22 | 2.87 | 15.24 |
| HPK1 | 19.62 | 10.86 | 6.19 | 15.24 |
| TGFβR2 | 25.93 | 4.50 | 15.16 | 15.21 |
| YES | 15.40 | 14.65 | 0.53 | 15.03 |
| CDK4/CycD3 | 13.64 | 15.63 | 1.40 | 14.64 |
| AMPKα1/β2/γ1 | 22.15 | 6.99 | 10.72 | 14.57 |
| MINK | 13.24 | 15.85 | 1.84 | 14.54 |
| SIK | 15.34 | 12.04 | 2.33 | 13.69 |
| PAK5 | 13.92 | 13.18 | 0.52 | 13.55 |
| AMPKα1/β1/γ3 | 22.65 | 4.45 | 12.87 | 13.55 |
| TSSK3 | 16.04 | 11.04 | 3.53 | 13.54 |
| MER | 11.38 | 15.20 | 2.70 | 13.29 |
| RIPK2 | 7.81 | 18.76 | 7.74 | 13.29 |
| DCAMKL2 | 16.81 | 9.43 | 5.22 | 13.12 |
| MAPKAPK2 | 17.30 | 8.93 | 5.91 | 13.12 |
| CK1δ | 8.82 | 17.39 | 6.06 | 13.10 |
| PKR | 13.54 | 12.59 | 0.67 | 13.07 |
| P38α | 7.70 | 18.35 | 7.53 | 13.03 |
| CHK2 | 8.24 | 17.68 | 6.67 | 12.96 |
| MST2 | 17.64 | 8.24 | 6.65 | 12.94 |
| NEK9 | 17.46 | 8.34 | 6.45 | 12.90 |
| YSK1 | 21.58 | 4.14 | 12.33 | 12.86 |
| GPRK6 | -3.18 | 28.89 | 22.68 | 12.85 |
| CDK9/CycT1 | 17.59 | 8.09 | 6.72 | 12.84 |
| PKCβ1 | 9.91 | 15.66 | 4.07 | 12.79 |
| SIK3 | 12.80 | 12.28 | 0.36 | 12.54 |
| TLK1 | 17.93 | 6.77 | 7.89 | 12.35 |
| MARK1 | 13.08 | 11.58 | 1.06 | 12.33 |
| SLK | 13.06 | 11.32 | 1.23 | 12.19 |
| GSK3α | 14.38 | 9.82 | 3.22 | 12.10 |
| MST3 | 7.18 | 16.83 | 6.82 | 12.00 |
| PKD2 | 18.75 | 5.20 | 9.58 | 11.98 |
| MST1 | 12.87 | 10.91 | 1.39 | 11.89 |
| BTK T316A | 11.44 | 12.32 | 0.62 | 11.88 |
| TXK | 11.06 | 12.37 | 0.92 | 11.72 |
| NEK1 | 9.40 | 13.73 | 3.06 | 11.57 |
| PKACγ | 9.00 | 14.02 | 3.55 | 11.51 |
| CDK6/CycD1 | 11.70 | 11.20 | 0.35 | 11.45 |
| WNK3 | 10.03 | 12.75 | 1.92 | 11.39 |
| RSK1 | 14.29 | 8.09 | 4.38 | 11.19 |
| CaMK2α | 10.62 | 11.73 | 0.79 | 11.17 |
| LATS2 | 17.11 | 5.03 | 8.54 | 11.07 |
| AMPKα2/β2/γ1 | 14.79 | 7.31 | 5.29 | 11.05 |
| PDGFRα | 9.89 | 12.07 | 1.54 | 10.98 |
| MAP2K2 | 16.88 | 4.93 | 8.45 | 10.90 |
| OSR1 | 6.66 | 15.04 | 5.92 | 10.85 |
| ULK2 | 17.75 | 3.81 | 9.86 | 10.78 |
| BRSK1 | 9.26 | 11.76 | 1.76 | 10.51 |
| CRIK | 11.99 | 8.98 | 2.13 | 10.48 |
| HIPK3 | 14.22 | 6.65 | 5.36 | 10.43 |
| GSK3β | 19.97 | 0.83 | 13.54 | 10.40 |
| FER | 10.05 | 10.64 | 0.42 | 10.34 |
| BTK C481S | 9.84 | 10.80 | 0.68 | 10.32 |
| NEK4 | 10.20 | 10.30 | 0.07 | 10.25 |
| LOK | 11.98 | 8.51 | 2.45 | 10.24 |
| AMPKα1/β1/γ2 | 20.46 | 0.00 | 14.47 | 10.23 |
| CK1γ2 | 3.82 | 16.04 | 8.64 | 9.93 |
| JAK1 | 13.60 | 6.19 | 5.24 | 9.90 |
| Erk5 | 9.67 | 9.91 | 0.17 | 9.79 |
| CDK7/CCNH/MNAT1 | 12.02 | 7.56 | 3.16 | 9.79 |
| CaMK1δ | 5.79 | 13.72 | 5.60 | 9.76 |
| BTK T474S | 0.93 | 18.40 | 12.35 | 9.66 |
| DYRK2 | 9.92 | 8.94 | 0.69 | 9.43 |
| MET Y1230D | 9.25 | 9.52 | 0.19 | 9.38 |
| CaMK1β | 4.61 | 14.11 | 6.72 | 9.36 |
| EGFR D746-750 T790M L858R | 9.66 | 9.02 | 0.45 | 9.34 |
| MAP2K3 | 12.43 | 5.83 | 4.67 | 9.13 |
| CLK3 | 9.72 | 8.37 | 0.95 | 9.05 |
| PLK2 | 6.03 | 12.05 | 4.25 | 9.04 |
| NPM1-ALK | 20.45 | -2.40 | 16.16 | 9.03 |
| NIM1K | 5.63 | 12.32 | 4.73 | 8.98 |
| DDR2 | 9.03 | 8.38 | 0.46 | 8.71 |
| CDK3/CycE1 | 10.65 | 6.45 | 2.97 | 8.55 |
| NEK3 | 13.41 | 3.58 | 6.95 | 8.50 |
| SGK | 17.72 | -0.88 | 13.15 | 8.42 |
| NDR1 | 18.20 | -1.39 | 13.85 | 8.41 |
| MNK2 | 10.34 | 6.40 | 2.78 | 8.37 |
| PKCα | 14.95 | 1.54 | 9.48 | 8.24 |
| TTBK1 | -0.92 | 17.30 | 12.88 | 8.19 |
| CDK2/CycE1 | 17.42 | -1.10 | 13.09 | 8.16 |
| DYRK1A | 7.84 | 8.47 | 0.44 | 8.16 |
| MLK1 | 13.92 | 2.17 | 8.31 | 8.04 |
| MYO3β | 8.37 | 7.61 | 0.54 | 7.99 |
| EGFR d746-750 C797S | 6.86 | 8.50 | 1.16 | 7.68 |
| PKN2 | 15.32 | 0.04 | 10.80 | 7.68 |
| NDR2 | 5.44 | 9.87 | 3.14 | 7.66 |
| EPHA6 | 12.09 | 3.17 | 6.31 | 7.63 |
| MARK3 | 7.24 | 8.00 | 0.53 | 7.62 |
| RSK4 | 6.01 | 9.11 | 2.20 | 7.56 |
| CDK5/p35NCK | 11.51 | 3.28 | 5.82 | 7.40 |
| p38β | 8.44 | 6.01 | 1.72 | 7.22 |
| p70S6K | 11.27 | 3.08 | 5.79 | 7.17 |
| PRKCH | 5.56 | 8.68 | 2.21 | 7.12 |
| CDK12/CycK | 3.56 | 10.59 | 4.97 | 7.08 |
| IKKα | 8.50 | 5.49 | 2.13 | 6.99 |
| AurB | 5.04 | 8.90 | 2.73 | 6.97 |
| AKT1 | 3.31 | 10.50 | 5.09 | 6.91 |
| BUB1/BUB3 | 7.94 | 5.38 | 1.81 | 6.66 |
| AMPKα2/β1/γ3 | 6.55 | 6.73 | 0.13 | 6.64 |
| BTK | 8.63 | 4.66 | 2.81 | 6.64 |
| PKN1 | 7.24 | 6.03 | 0.86 | 6.63 |
| TAOK2 | 8.44 | 4.80 | 2.58 | 6.62 |
| AMPKα1/β2/γ3 | 8.41 | 4.75 | 2.58 | 6.58 |
| MST4 | 9.30 | 3.71 | 3.95 | 6.50 |
| AMPKα2/β1/γ2 | 11.44 | 1.07 | 7.33 | 6.26 |
| MET D1228N | 7.33 | 4.97 | 1.67 | 6.15 |
| p38γ | 5.24 | 6.83 | 1.12 | 6.03 |
| COT | 8.63 | 3.24 | 3.81 | 5.94 |
| PIM1 | 9.00 | 2.85 | 4.34 | 5.93 |
| PAK4 | 5.57 | 6.26 | 0.49 | 5.92 |
| MARK4 | 2.43 | 9.22 | 4.80 | 5.83 |
| SGK3 | 1.67 | 9.91 | 5.82 | 5.79 |
| PRKCD | 7.85 | 3.61 | 3.00 | 5.73 |
| PDGFRα T674I | 4.19 | 7.11 | 2.06 | 5.65 |
| CDK1/CycE1 | 8.16 | 3.06 | 3.60 | 5.61 |
| CDK1/CycA2 | 9.31 | 1.80 | 5.31 | 5.55 |
| MRCKα | -6.10 | 17.05 | 16.37 | 5.48 |
| MAP2K5 | -3.33 | 14.15 | 12.36 | 5.41 |
| CK1γ3 | 9.21 | 1.57 | 5.40 | 5.39 |
| ABL2 | 2.12 | 8.56 | 4.55 | 5.34 |
| HIPK4 | 5.74 | 4.87 | 0.62 | 5.31 |
| EPHA4 | -4.76 | 15.24 | 14.14 | 5.24 |
| PYK2 | 14.49 | -4.01 | 13.08 | 5.24 |
| SRM | 7.80 | 2.19 | 3.97 | 4.99 |
| MRCKβ | 16.25 | -6.33 | 15.96 | 4.96 |
| CDK2/CycA2 | 6.34 | 3.55 | 1.97 | 4.94 |
| TSSK1 | 5.80 | 3.53 | 1.61 | 4.66 |
| ZAP70 | 9.28 | -0.01 | 6.57 | 4.63 |
| CDK9/CycT2 | 4.49 | 4.77 | 0.19 | 4.63 |
| DYRK4 | 8.94 | 0.30 | 6.11 | 4.62 |
| PRKCE | 6.97 | 2.09 | 3.45 | 4.53 |
| HER4 | 5.03 | 3.83 | 0.85 | 4.43 |
| IKKβ | 2.53 | 5.78 | 2.30 | 4.16 |
| CDK16/CycY | 5.63 | 2.65 | 2.11 | 4.14 |
| STK33 | 6.81 | 1.28 | 3.91 | 4.05 |
| CAMKK2 | 0.11 | 7.92 | 5.52 | 4.02 |
| JNK1 | 7.46 | 0.17 | 5.15 | 3.82 |
| GAK | 6.69 | 0.83 | 4.14 | 3.76 |
| AMPKα1/β1/γ1 | 9.08 | -1.73 | 7.65 | 3.68 |
| MET Y1230A | 5.65 | 1.62 | 2.85 | 3.64 |
| ERN1 | 0.41 | 6.65 | 4.41 | 3.53 |
| AXL | 4.07 | 3.00 | 0.75 | 3.53 |
| ULK1 | 5.48 | 1.44 | 2.86 | 3.46 |
| EGFR L858R | 3.84 | 3.07 | 0.55 | 3.45 |
| EGFR d746-750 T790M C797S | -2.39 | 9.13 | 8.15 | 3.37 |
| ULK3 | 6.56 | -0.36 | 4.90 | 3.10 |
| CDK13/CycK | 7.39 | -1.21 | 6.08 | 3.09 |
| DAPK1 | 5.61 | 0.56 | 3.57 | 3.08 |
| ALK | 7.09 | -1.24 | 5.89 | 2.92 |
| Erk2 | 3.56 | 2.13 | 1.01 | 2.84 |
| PRKCZ | 2.96 | 2.54 | 0.30 | 2.75 |
| EGFR L861Q | -0.39 | 5.87 | 4.42 | 2.74 |
| PRKCI | 1.27 | 4.11 | 2.01 | 2.69 |
| EPHA2 | -4.27 | 9.59 | 9.80 | 2.66 |
| PKCβ2 | 1.77 | 3.53 | 1.25 | 2.65 |
| IRAK4 | 5.10 | 0.07 | 3.56 | 2.59 |
| BRAF V600E | -2.22 | 7.31 | 6.74 | 2.54 |
| EGFR A763_Y764insFQEA | 4.17 | 0.89 | 2.32 | 2.53 |
| AMPKα2/β1/γ1 | 6.66 | -1.79 | 5.97 | 2.43 |
| PAK2 | 2.92 | 1.94 | 0.70 | 2.43 |
| AKT2 | 1.95 | 2.78 | 0.58 | 2.37 |
| LIMK1 | -2.49 | 7.12 | 6.80 | 2.32 |
| ROS | 0.65 | 3.86 | 2.27 | 2.25 |
| PASK | 4.23 | 0.26 | 2.81 | 2.24 |
| RON | 3.64 | 0.80 | 2.01 | 2.22 |
| MAP3K5 | 0.17 | 4.23 | 2.87 | 2.20 |
| WNK2 | 6.15 | -1.84 | 5.65 | 2.15 |
| EPHA5 | -9.58 | 13.79 | 16.52 | 2.10 |
| EPHA8 | 0.97 | 3.13 | 1.53 | 2.05 |
| CDK5/p25NCK | 8.18 | -4.13 | 8.70 | 2.03 |
| DMPK1 | 10.64 | -6.69 | 12.26 | 1.97 |
| PKACα | -5.16 | 9.10 | 10.08 | 1.97 |
| PKD3 | 4.27 | -0.66 | 3.49 | 1.81 |
| PRKCQ | 1.00 | 2.57 | 1.11 | 1.79 |
| PHKG1 | -1.28 | 4.79 | 4.30 | 1.76 |
| BMX | 0.08 | 3.26 | 2.25 | 1.67 |
| TEC | 2.63 | 0.56 | 1.46 | 1.59 |
| MNK1 | -5.38 | 8.51 | 9.82 | 1.56 |
| EGFR D770_N771insNPG | -3.41 | 6.36 | 6.91 | 1.47 |
| EPHB1 | 1.41 | 1.53 | 0.09 | 1.47 |
| EGFR V769-D770 insGE | 3.29 | -0.41 | 2.62 | 1.44 |
| IRAK1 | -2.93 | 5.57 | 6.01 | 1.32 |
| BMPR2 | 0.41 | 2.18 | 1.25 | 1.30 |
| DCAMKL1 | 15.22 | -12.67 | 19.72 | 1.27 |
| PAK1 | 2.42 | -0.09 | 1.77 | 1.17 |
| CDC7/ASK | 0.12 | 2.18 | 1.46 | 1.15 |
| CaMK2δ | 4.25 | -2.03 | 4.44 | 1.11 |
| EGFR D746-750 T790M C797S L858R | -2.48 | 4.54 | 4.97 | 1.03 |
| CLK2 | -1.22 | 3.15 | 3.09 | 0.96 |
| JAK3 | -0.42 | 2.18 | 1.84 | 0.88 |
| MET | 7.54 | -6.37 | 9.84 | 0.58 |
| PIM3 | -6.88 | 7.95 | 10.49 | 0.54 |
| NIK | 2.68 | -1.77 | 3.14 | 0.45 |
| FLT3 D835Y | 13.44 | -12.59 | 18.41 | 0.43 |
| EGFR L858R T790M | 0.28 | 0.43 | 0.11 | 0.36 |
| CaMK2γ | 0.03 | 0.67 | 0.45 | 0.35 |
| CK1α | 1.42 | -0.75 | 1.53 | 0.34 |
| P70S6Kβ | -4.03 | 4.50 | 6.03 | 0.24 |
| EGFR C797S L858R | -0.62 | 0.69 | 0.92 | 0.04 |
| EGFR T790M C797S | 0.56 | -0.54 | 0.78 | 0.01 |
| ALK2 | 5.59 | -5.59 | 7.91 | 0.00 |
| GPRK4 | 1.72 | -1.75 | 2.45 | -0.01 |
| EGFR | -0.37 | 0.32 | 0.49 | -0.02 |
| EGFR T790M | 0.66 | -0.71 | 0.97 | -0.02 |
| AurC | -1.38 | 0.97 | 1.66 | -0.20 |
| EGFR C797S | -2.57 | 2.16 | 3.35 | -0.21 |
| HIPK1 | 3.89 | -4.54 | 5.96 | -0.33 |
| CDK1/CycB1 | 0.23 | -1.05 | 0.90 | -0.41 |
| PLK3 | 1.46 | -2.44 | 2.75 | -0.49 |
| PKACβ | 7.38 | -8.43 | 11.18 | -0.53 |
| WNK1 | -5.88 | 4.80 | 7.55 | -0.54 |
| EGFR d746-750 C797G | -7.40 | 6.10 | 9.54 | -0.65 |
| QIK | 7.11 | -8.74 | 11.21 | -0.81 |
| ROCK1 | -0.15 | -1.53 | 0.97 | -0.84 |
| MAP3K19 | 1.09 | -2.94 | 2.85 | -0.92 |
| BRSK2 | 1.32 | -3.28 | 3.25 | -0.98 |
| EGFR T790M C797S L858R | 2.23 | -4.25 | 4.58 | -1.01 |
| JAK2 | -1.93 | -0.84 | 0.77 | -1.38 |
| RSK3 | -3.86 | 1.07 | 3.49 | -1.39 |
| BRAF | -3.88 | 1.09 | 3.52 | -1.39 |
| FRK | 2.53 | -5.79 | 5.89 | -1.63 |
| AKT3 | 0.67 | -3.94 | 3.26 | -1.64 |
| EGFR d746-750 | -2.27 | -1.06 | 0.86 | -1.67 |
| SRC | -13.35 | 9.91 | 16.44 | -1.72 |
| Erk7 | 3.70 | -7.29 | 7.77 | -1.80 |
| PBK | -3.43 | -0.46 | 2.10 | -1.94 |
| MAP3K4 | -0.97 | -3.01 | 1.44 | -1.99 |
| PRKX | -0.36 | -4.03 | 2.60 | -2.19 |
| BARK1 | -6.36 | 1.97 | 5.88 | -2.20 |
| TTBK2 | -3.30 | -1.63 | 1.18 | -2.47 |
| RET V804E | -7.73 | 2.75 | 7.41 | -2.49 |
| LYNa | -3.99 | -1.20 | 1.97 | -2.59 |
| PKD1 | -0.96 | -4.37 | 2.41 | -2.66 |
| FES | 0.58 | -6.35 | 4.90 | -2.89 |
| EPHA3 | -1.19 | -5.70 | 3.18 | -3.45 |
| IGF1R | -3.14 | -3.95 | 0.57 | -3.55 |
| PHKG2 | -3.75 | -3.36 | 0.27 | -3.55 |
| MSK2 | 5.42 | -12.58 | 12.73 | -3.58 |
| TBK1 | -5.87 | -1.72 | 2.93 | -3.79 |
| EGFR D746-750 T790M | -4.08 | -3.63 | 0.32 | -3.85 |
| SGK2 | -11.71 | 3.76 | 10.94 | -3.98 |
| PAK6 | 1.17 | -9.20 | 7.33 | -4.02 |
| CDK4/CycD1 | -4.96 | -3.26 | 1.20 | -4.11 |
| PKN3 | -7.94 | -0.59 | 5.20 | -4.26 |
| KIT D816V | -3.20 | -5.44 | 1.58 | -4.32 |
| CLK1 | -0.39 | -8.27 | 5.58 | -4.33 |
| MAPKAPK3 | -3.28 | -5.61 | 1.65 | -4.45 |
| NLK | -5.65 | -3.25 | 1.70 | -4.45 |
| MAP3K1 | -2.79 | -6.68 | 2.75 | -4.73 |
| KIT V559D | -0.74 | -8.75 | 5.66 | -4.75 |
| TAOK1 | -3.26 | -7.03 | 2.67 | -5.14 |
| CHK1 | -2.78 | -7.65 | 3.45 | -5.21 |
| CDK9/CycK | -4.16 | -6.78 | 1.85 | -5.47 |
| GSG2 | -9.94 | -1.05 | 6.29 | -5.50 |
| ABL1 H396P | -0.43 | -10.95 | 7.44 | -5.69 |
| CDK18/CycY | -4.10 | -7.30 | 2.26 | -5.70 |
| LCK | -13.15 | 0.74 | 9.82 | -6.20 |
| CSK | -7.01 | -6.53 | 0.34 | -6.77 |
| WEE1 | -0.70 | -12.94 | 8.65 | -6.82 |
| PIM2 | -3.59 | -10.96 | 5.22 | -7.28 |
| MARK2 | -10.24 | -5.12 | 3.62 | -7.68 |
| AMPKα1/β2/γ2 | -9.92 | -5.56 | 3.08 | -7.74 |
| TLK2 | -2.10 | -13.87 | 8.32 | -7.98 |
| TAOK3 | -5.46 | -10.56 | 3.61 | -8.01 |
| GPRK7 | -10.70 | -5.62 | 3.59 | -8.16 |
| DMPK2 | -8.14 | -8.54 | 0.29 | -8.34 |
| CaMK2β | -5.15 | -12.24 | 5.01 | -8.69 |
| TYK2 | -6.35 | -12.19 | 4.14 | -9.27 |
| BARK2 | -11.01 | -7.59 | 2.41 | -9.30 |
| PAK3 | -14.45 | -4.59 | 6.97 | -9.52 |
| ROCK2 | -14.20 | -5.64 | 6.05 | -9.92 |
| AAK1 | -12.24 | -8.17 | 2.88 | -10.20 |
| EPHB3 | -9.42 | -11.64 | 1.56 | -10.53 |
| LATS1 | -12.64 | -9.53 | 2.20 | -11.08 |
| TYRO3 | -8.18 | -14.44 | 4.43 | -11.31 |
| ABL1 | -2.93 | -20.08 | 12.13 | -11.50 |
| PLK1 | -11.29 | -11.73 | 0.31 | -11.51 |
| FYN [isoform b] | -18.79 | -4.28 | 10.26 | -11.53 |
| PIK3CA | -13.43 | -10.63 | 1.98 | -12.03 |
| EPHB2 | -4.82 | -19.48 | 10.36 | -12.15 |
| PIK3CB | -11.23 | -14.32 | 2.19 | -12.77 |
| EPHA1 | -8.35 | -18.73 | 7.34 | -13.54 |
| EPHB4 | -10.51 | -17.14 | 4.69 | -13.83 |
| PGK | -14.74 | -14.18 | 0.39 | -14.46 |
| ITK | -11.08 | -17.96 | 4.87 | -14.52 |
| PIK3CD | -16.84 | -15.08 | 1.24 | -15.96 |
| BMP2K | -19.72 | -12.64 | 5.01 | -16.18 |
| SYK | -21.97 | -20.04 | 1.37 | -21.01 |
| HER2 | -55.09 | -64.33 | 6.53 | -59.71 |

The ^1^H, ^13^C NMR, HPLC and HRMS data of compounds 8a-s

The ^1^H, ^13^C NMR, HPLC and HRMS traces of compound **8a**.

The ^1^H, ^13^C NMR, HPLC and HRMS traces of compound **8b**.

The ^1^H, ^13^C NMR, HPLC and HRMS traces of compound **8c**.

The ^1^H, ^13^C NMR, HPLC and HRMS traces of compound **8d**.

The ^1^H, ^13^C NMR, HPLC and HRMS traces of compound **8e**.

The ^1^H, ^13^C NMR, HPLC and HRMS traces of compound **8f**.

The ^1^H, ^13^C NMR, HPLC and HRMS traces of compound **8g**.

The ^1^H, ^13^C NMR, HPLC and HRMS traces of compound **8h**.

The ^1^H, ^13^C NMR, HPLC and HRMS traces of compound **8i**.

The ^1^H, ^13^C NMR, HPLC and HRMS traces of compound **8j**.

The ^1^H, ^13^C NMR, HPLC and HRMS traces of compound **8k**.

The ^1^H, ^13^C NMR, HPLC and HRMS traces of compound **8l**.

The ^1^H, ^13^C NMR, HPLC and HRMS traces of compound **8m**.

The ^1^H, ^13^C NMR, HPLC and HRMS traces of compound **8n**

The ^1^H, ^13^C NMR, HPLC and HRMS traces of compound **8o**.

The ^1^H, ^13^C NMR, HPLC and HRMS traces of compound **8p**.

The ^1^H, ^13^C NMR, HPLC and HRMS traces of compound **8q**.

The ^1^H, ^13^C NMR, HPLC and HRMS traces of compound **8r**.

The ^1^H, ^13^C NMR, HPLC and HRMS traces of compound **8s**.
